# Accuracy of tomographic and biomechanical parameters in detecting unilateral post-LASIK keratoectasia and fellow eyes

**DOI:** 10.3389/fbioe.2023.1181117

**Published:** 2023-06-02

**Authors:** Kaili Yang, Qi Fan, Liyan Xu, Yuwei Gu, Chenjiu Pang, Shengwei Ren

**Affiliations:** Henan Provincial People’s Hospital, Henan Eye Hospital, Henan Eye Institute, People’s Hospital of Zhengzhou University, Henan University People’s Hospital, Zhengzhou, China

**Keywords:** fellow eye, corneal biomechanics, diagnostic test, unilateral post-LASIK keratectasia, corneal tomographic parameters

## Abstract

**Background:** Patients with unilateral post-LASIK keratectasia (KE) have clinical ectasia in one eye but not in the fellow eye. As serious complications, these cases are rarely reported but are worth investigating. This study aimed to explore the characteristics of unilateral KE and the accuracy of corneal tomographic and biomechanical parameters to detect KE and distinguish fellow eyes from control eyes.

**Methods:** The study analyzed 23 KE eyes, 23 KE fellow eyes, and 48 normal eyes from age- and sex-matched patients who had undergone LASIK. The Kruskal–Wallis test and further paired comparisons were performed to compare the clinical measurements of the three groups. The receiver operating characteristic curve was used to evaluate the ability to distinguish KE and fellow eyes from the control eyes. Binary logistic regression with the forward stepwise method was performed to produce a combined index, and the DeLong test was used to compare the discriminability difference of the parameters.

**Results:** Males accounted for 69.6% of patients with unilateral KE. The duration between corneal surgery and the onset of ectasia ranged from 4 months to 18 years, with a median time of 10 years. The KE fellow eye had a higher posterior evaluation (PE) value than the control eyes (5 vs. 2, *p* = 0.035). Diagnostic tests showed that PE, posterior radius of curvature (3 mm), anterior evaluation (FE), and Corvis biomechanical index–laser vision correction (CBI-LVC) were sensitive indicators for distinguishing KE in the control eyes. The ability of PE to detect the KE fellow eye from the control eye was 0.745 (0.628 and 0.841), with 73.91% sensitivity and 68.75% specificity at a cut-off value of 3. The ability of a combined index, constructed using PE and FE, to distinguish fellow eyes of KE from controls was 0.831 (0.723 and 0.909), which was higher than that of PE and FE individually (*p* < 0.05).

**Conclusion:** The fellow eyes of patients with unilateral KE had significantly higher PE values than control eyes, and a combination of PE and FE enhanced this differentiation in a Chinese population. More attention should be paid to the long-term follow-up of patients after LASIK and to be wary of the occurrence of early KE.

## 1 Introduction

Laser-assisted *in situ* keratomileusis (LASIK) is an effective refractive surgery performed worldwide (Sandoval et al., 2016). On average, 1,608,880 corneal refractive surgery procedures were conducted per year between 1991 and 2015 (Moshirfar et al., 2021). With an increasing number of surgeries being performed, surgical complications, which are often irreversible, have attracted attention in recent years (Bohac et al., 2018). Post-LASIK keratectasia (KE) after corneal refractive surgery is a serious complication that was first reported by Seiler et al. (1998). Previous studies have reported that the incidence of KE after LASIK ranged from 0.033% to 0.9%, with a postoperative follow-up period of 2–10 years (Pallikaris et al., 2001; Randleman et al., 2003; Moshirfar et al., 2014; Santhiago et al., 2016; Bohac et al., 2018; Ambrosio, 2019). Similar to primary keratoconus (KC), KE is characterized by thinning and bulging of the cornea, loss of visual acuity, and irregular clinical astigmatism (Ambrosio et al., 2010). Global consensus stated that true unilateral KC is nonexistent, and the fellow normal eye of asymmetric patients may develop KC (Gomes et al., 2015). The justification is related to KC being considered a genetic-related condition (Santodomingo-Rubido et al., 2022). Furthermore, several studies have reported that the corneal shape and biomechanical parameters of the KC fellow eye were different from KC and normal control eyes (Bae et al., 2014; Degirmenci et al., 2019; Koc et al., 2019). Therefore, it is worth investigating whether the same phenomenon exists in patients with unilateral KE who underwent bilateral corneal refractive surgery.

Unilateral KE, iatrogenic secondary corneal ectasia, could occur due to a purely mechanical process (Ambrosio, 2019; Salomão et al., 2021). While the prevalence is relatively low, the incidence of KE is increasing in clinical practice (Bohac et al., 2018). The characteristics of patients with unilateral KE have important research value, but case studies are rarely reported. Although slit-lamp examination has traditionally been used to diagnose KE, corneal tomographic and biomechanical parameters, which are important for the early diagnosis of corneal diseases, are gradually being used to identify KE. Ueki et al. (2018) and Yang et al. (2020) reported that central corneal thickness exhibited no significant difference between normal post-LASIK eyes and KE eyes, but the radius and deflection amplitude at the highest concavity were significantly different. In addition, our previous study showed that KE eyes exhibited a lower stiffness parameter at first applanation value than did normal LASIK eyes and higher values of maximum inverse radius, deformation amplitude (DA) ratio max (2 mm), pachy slope, DA ratio max (1 mm), and integrated radius (Yang et al., 2020). However, research on the fellow eyes of patients with unilateral KE is limited, and the accuracy of corneal tomographic and biomechanical parameters to distinguish clinical KE and fellow eyes from control eyes has not been reported. Thus, the current study aimed to investigate the characteristics of unilateral KE in a Chinese population and further explore the ability of corneal tomographic and biomechanical parameters to distinguish KE and fellow eyes from control eyes to provide a reference for diagnosing KE early.

## 2 Materials and methods

### 2.1 Study participants

Patients with unilateral KE who were referred to Henan Eye Hospital between April 2018 and January 2023 were recruited for this retrospective analysis. The diagnostic criteria for unilateral KE were as follows: 1) eyes that underwent bilateral LASIK for myopia and myopic astigmatism; 2) KE eyes that have the presence of certain signs on corneal tomography (such as displacement of the corneal apex, a decrease in pachymetry, an asymmetric tomographic pattern, and posterior elevation (PE) values above 16) or with abnormal indications of slit-lamp examination (central corneal thinning, conical protrusion, Vogt’s striae, Munson’s signs, or Fleischer’s ring) (Chan et al., 2018; Yang et al., 2020), while the KE fellow eyes have no aforementioned signs. The inclusion criteria for the control eyes were as follows: 1) eyes that underwent corneal refractive surgery at least 1 year prior; 2) eyes with corrected distance visual acuity with logMAR ≤0.1; 3) eyes with no detectable disease; and 4) eyes that were age- and sex-matched. In contrast, eyes in contact with rigid contact lenses within the past 4 weeks and soft contact lenses within the past 2 weeks and patients with severe uncontrolled diabetes, other eye conditions (e.g., cataract and glaucoma), and a history of previous eye surgeries (except refractive surgeries) were excluded. Overall, 23 patients with unilateral KE (23 KE eyes and 23 fellow eyes) and 48 control eyes were included in the analysis. The study was approved by the Institutional Review Board of Henan Eye Hospital [ethical approval number HNEECKY-2019 (5)], and written informed consent was obtained from all the participants.

### 2.2 Examinations

All patients underwent the following clinical examinations (Yang et al., 2021): autorefraction (Topcon KR-800), a standard logarithmic visual acuity chart to obtain corrected distance visual acuity, slit-lamp examination (Vogt’s striae, Fleischer’s ring, Munson’s sign, and corneal scarring), and corneal tomography and biomechanical parameter. Experienced operators conducted the measurements between 9:00 and 17:00.

Corneal tomography parameters were measured using Pentacam HR (Oculus, Wetzlar, Germany), which uses a rotating high-resolution camera to analyze the eye to assess the anterior and posterior surfaces of the cornea (de Luis Eguileor et al., 2018) (Hwang et al., 2018). The findings with a high-quality factor were recorded for each eye, and the following parameters were analyzed: (1) the central 3.0 mm of the anterior and posterior corneal surfaces in terms of flat, steep, and mean keratometries; (2) the maximum keratometry of the anterior corneal surface; (3) the corneal thickness at the pachy apex, the center of the pupil, and the thinnest point of the cornea; (4) topometric screening indices containing the index of surface variation, index of vertical asymmetry, keratoconus index (KI), central KI (CKI), index of height asymmetry, and index of height decentration; (5) the thinnest corneal point (anterior evaluation [FE] and PE values); and (6) Belin–Ambrósio display indices (D). In addition, the flap thickness was measured manually, and N1–N4 and N6–N23 patients were measured through CASIA SS-1000, and the N5 patient was measured through Visante OCT (Zeiss).

Corneal biomechanics were obtained using Corneal Visualization Scheimpflug Technology (Corvis-ST, Oculus, Wetzlar, Germany), which is a non-contact tonometer. Decompensation of biomechanical properties is the initiating element of ectasia progression (Vinciguerra et al., 2016a; Vinciguerra et al., 2017). The instrument uses Scheimpflug images of the anterior segment at a rate of 4,330 frames/s and can obtain corneal biomechanical parameters through three phases: first applanation, the highest concavity, and second applanation (Jedzierowska and Koprowski, 2019; Yang et al., 2019). The applanation time, velocity, radius (calculated during the concave phase of the deformation response) (Vinciguerra et al., 2016b), DA, deflection length, deflection amplitude (DLA), deflection area (DLAr), and delta arc length were recorded. In addition, intraocular pressure, biomechanical corrected intraocular pressure, central corneal thickness, peak distance, radius, and whole eye movement are presented. New parameters, such as DA ratio max (1 mm), DA ratio max (2 mm)^27^, pachy slope, max inverse radius, integrated radius, Ambrósio’s relational thickness horizontal profile, and stiffness parameter at first applanation were added using the updated software (software number 1.5r1902) (Vinciguerra et al., 2016a), and Corvis biomechanical index–laser vision correction (CBI-LVC) was calculated on the basis of a logistic regression formula (Vinciguerra et al., 2021).

### 2.3 Analytical tool and method

The quantitative data of the patients are presented as the median (M) and range (P25 and P75). The Kruskal–Wallis test was used to compare the differences among the control, KE, and fellow eyes, and further paired comparisons of the least significant difference were performed. The receiver operating characteristic curve was used to evaluate the ability of the parameters to distinguish clinical KE and the fellow eye from control eyes, and the area under the receiver operating characteristic curve (AUC) and 95% confidence interval (CI) were recorded. The combined model was constructed to improve the ability to detect the KE fellow eye, which used binary logistic regression with the forward stepwise method (*p* < 0.1 for retention in the model). The DeLong test was used to compare the differences in discriminability of the parameters. All data were analyzed using SPSS 23.0 and MedCalc 15.2.2, and *p* < 0.05 (two-tailed) was considered a statistically significant difference.

## 3 Results

### 3.1 Characteristics of demographic data

Table 1 shows the basic data from 23 patients with unilateral KE after corneal refractive surgery. The median age of KE diagnosis was 32 years, ranging from 18 to 38 years, and males accounted for 69.6% of all patients. The duration between corneal surgery and the onset of KE ranged from 4 months to 18 years, with a median of 10 years. Data related to the flap thickness were measured manually, and the flap thickness of the corresponding measurement positions was indicated by arrows in Supplementary Figure S1. No statistically significant differences in age, sex, and duration were found between the unilateral KE and control eyes (all *p* > 0.05).

**TABLE 1:** Basic data of 23 unilateral KE patients.

No.	Diagnosed age (y)	Sex	Duration time from surgery to diagnose ectasia	Ectasia eye	Flap thickness (right, µm)^a^	Flap thickness (left, µm)^a^
N1	25	M	5 years	Left	222	163
N2	33	M	10 years	Left	189	179
N3	37	F	11 years	Right	270	311
N4	28	M	10 years	Left	191	200
N5	34	F	13 years	Right	167	175
N6	23	M	8 months	Right	123	207
N7	19	M	4 months	Right	139	138
N8	35	M	13 years	Right	119	128
N9	35	M	10 years	Left	246	221
N10	38	F	14 years	Left	190	266
N11	33	M	15 years	Right	158	191
N12	32	M	13 years	Right	189	207
N13	25	M	5 years	Left	210	150
N14	38	F	18 years	Right	252	201
N15	31	F	2 years	Left	153	108
N16	25	M	4 years	Left	134	144
N17	32	F	12 years	Right	216	189
N18	34	M	9 years	Right	121	147
N19	35	M	11 years	Left	195	161
N20	20	M	15 months	Right	136	133
N21	18	M	1 year	Left	141	154
N22	33	M	10 years	Right	290	154
N23	31	M	10 years	Left	263	372

^a^ Data were measured manually, and the flap thickness of the corresponding measurement positions is indicated by arrows in Supplementary Figure S1.

### 3.2 Comparison of clinical measurements

**FIGURE 1:**
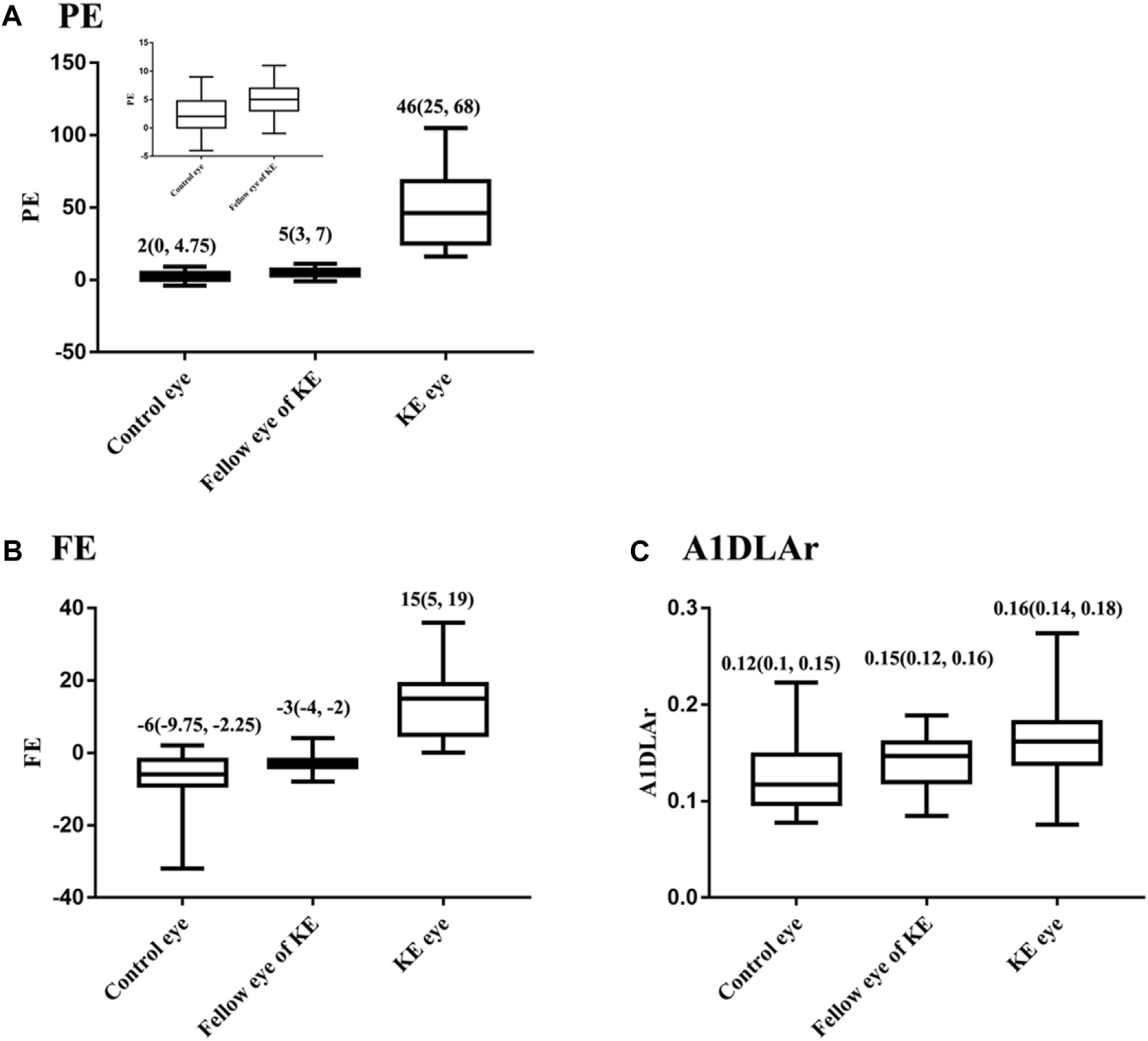
Distribution of PE, FE and among control, KE and the fellow eyes, M (P25, P75). **(A)** PE, **(B)** FE, **(C)** A1DLAr.

Corneal tomographic and biomechanical parameters were compared among the control, KE, and fellow eyes (Supplementary Tables S1, S2). Significant differences in clinical measurements were found between the KE and control eyes (all *p* < 0.05). Compared with the control eyes, KE fellow eyes had a higher PE value (5 vs. 2, *p* = 0.035, Figure 1A). At *p* < 0.1 level, the KE fellow eye had higher FE values (−3 vs. −6, *p* = 0.062, Figure 1B) and A1DLAr (0.15 vs. 0.12, *p* = 0.073, Figure 1C) values than the control eyes.

### 3.3 Ability of parameters to distinguish KE from control eyes

**FIGURE 2:**
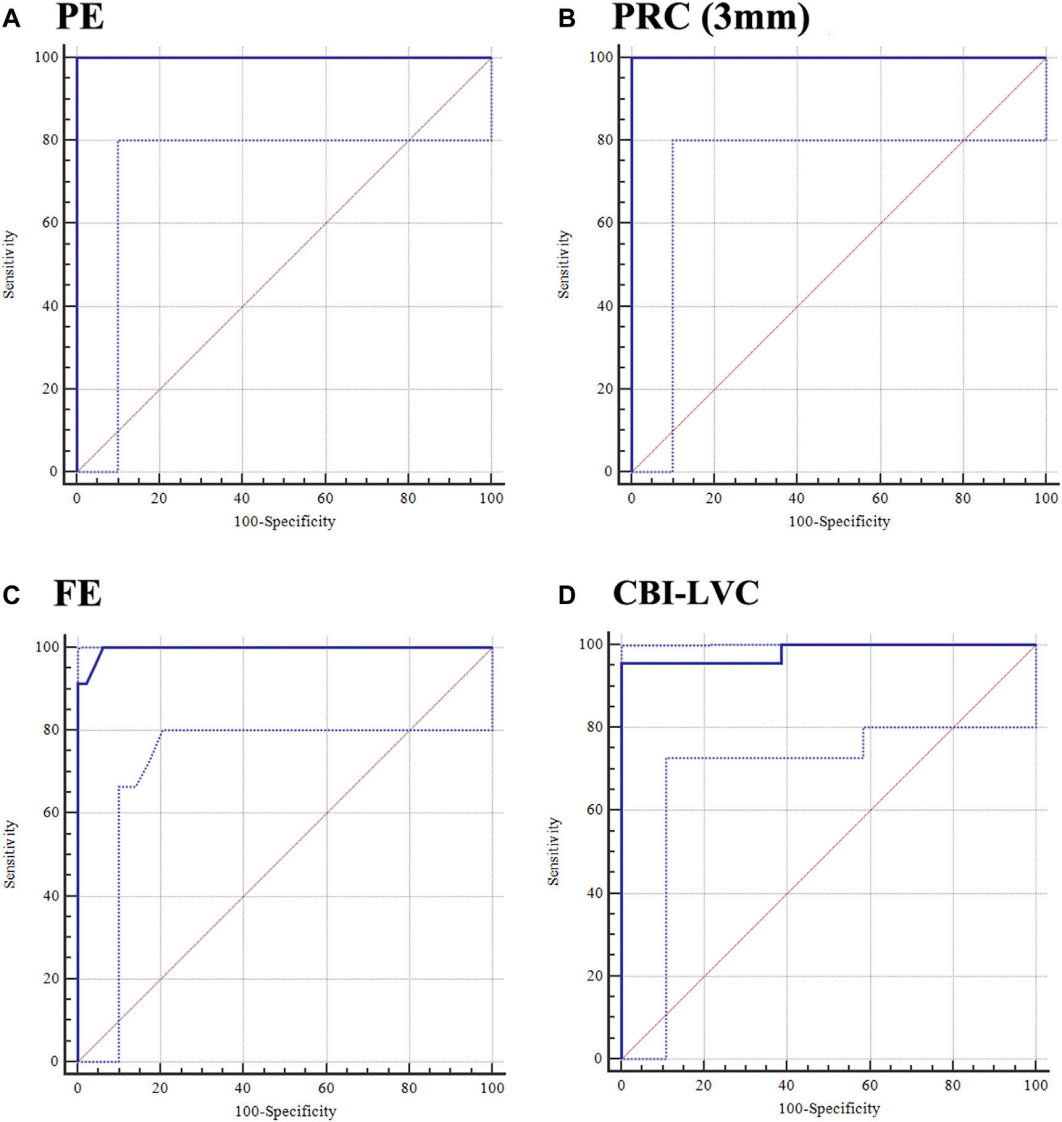
Ability of PE, PRC (3 mm), FE, and CBI-LVC in distinguishing the KE eye from the control eye. **(A)** PE; **(B)** PRC (3 mm); **(C)** FE; and **(D)** CBI-LVC.

Supplementary Table S3 shows the AUC of corneal tomographic parameters for distinguishing the KE eye from the control eye. Similar to PE, the posterior radius of curvature (PRC) (3 mm) correctly diagnosed KE eyes at a criterion value of 5.94 (Figures 2A, B). In addition, FE had a high Youden value in distinguishing KE from control eyes, with 100% sensitivity and 93.75% specificity at a cut-off value of −1 (Figure 2C). Among the corneal biomechanical parameters, the CBI-LVC was a relatively good parameter for detecting KE (0.983, 95% CI: 0.917-0.999), with 95.65% sensitivity and 100% specificity (Figure 2D; Supplementary Table S4).

### 3.4 Ability of parameters to distinguish KE fellow eyes from control eyes

**FIGURE 3:**
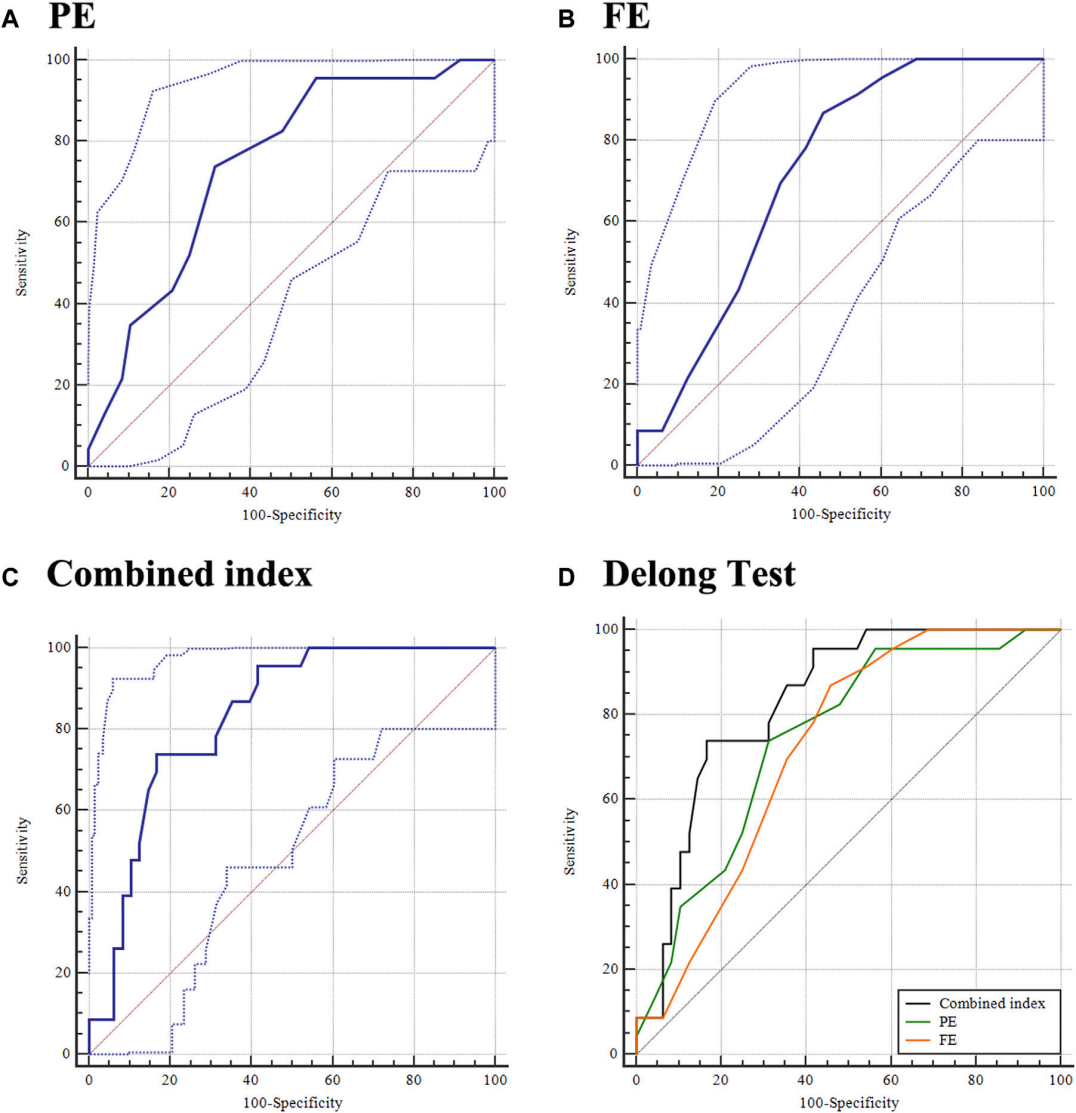
Ability of PE, FE, and combined index in distinguishing the fellow eye of KE from the control eye. **(A)** PE; **(B)** FE; **(C)** combined index; and **(D)** DeLong test.

The ability of PE to detect KE fellow eyes from control eyes was 0.745 (0.628 and 0.841), with 73.91% sensitivity and 68.75% specificity at a cut-off value of 3 (Table 2, Figure 3A). Furthermore, the AUCs (95% CIs) of FE (cut-off = −6, Figure 3B) and A1DLAr (cut off = 0.124) for diagnosing KE fellow eyes were 0.722 (0.603 and 0.822) and 0.687 (0.562 and 0.795), respectively.

**TABLE 2:** Ability of corneal tomographic parameters in distinguishing the fellow eye of the KE eye from control eyes.

Parameter	Cut-off	AUC (95% CI)	Youden index	Sensitivity (%)	Specificity (%)
PE	>3	0.745 (0.628, 0.841)	0.427	73.91	68.75
FE	>−6	0.722 (0.603, 0.822)	0.411	86.96	54.17
Combined index	>0.353	0.831 (0.723, 0.909)	0.573	73.91	83.33

Combined index = 0.343*PE + 0.264*FE–0.716.

Further logistic regression analysis included PE and FE in a combined model, in which the coefficients of PE and FE were 0.343 and 0.264, respectively (Supplementary Table S5 **,** all *p* < 0.05). The AUC (95% CI) of the combined index for distinguishing KE fellow eyes from control eyes was 0.831 (0.723 and 0.909), with 73.91% sensitivity and 83.33% at a cut-off value of 0.353 (Figure 3C). Further AUC pairwise comparisons are presented in Figure 3D, in which the combined index had a higher detection ability than the individual diagnosis ability of PE (AUC difference, 0.086; 95% CI: 0.007–0.164; *p* = 0.032) and FE (AUC difference: 0.109; 95% CI: 0.007–0.211; *p* = 0.037).

## 4 Discussion

The assessment of corneal shape and biomechanical parameters plays a vital role in evaluating eye conditions, and assessing the parameters of KE and fellow eyes helps diagnose early KE so as to avoid further vision loss (Sedaghat et al., 2018; Hocaoglu et al., 2020). The current study showed that PE was different between the KE fellow eyes and control eyes, and the combined index of PE and FE improved the ability to differentiate between the KE fellow and control eyes.

KE does not remain biomechanically stable during the postoperative period; however, KE eyes exhibit progressive central or inferior corneal steepening associated with stromal thinning, similar to that noted in KC eyes (Ueki et al., 2018; He et al., 2020). A previous study reported that central corneal thickness in normal post-LASIK and KE eyes decreased, which is related to the removal of corneal tissue, and softening of tissue would be expected because of structural alterations caused by severing tension-bearing lamellae (Lee et al., 2017; Ueki et al., 2018). Biomechanical instability of the cornea is linked to the development of KE, and unilateral secondary corneal ectasia can occur due to a purely mechanical process (Gomes et al., 2015; Bohac et al., 2018; Salomão et al., 2021). Several studies have reported the potential risk factors for developing KE, which include high myopia, thin cornea, other preoperatively suspicious KC symptoms, a high percentage of tissue altered, and low specific residual stromal bed thickness (Randleman et al., 2003; Santhiago et al., 2016). The preoperative data of the five patients were re-evaluated, revealing no abnormal tomographic map findings (Supplementary Table S6). Due to the long duration time from surgery to diagnose ectasia, the preoperative data and surgical data (level of correction, ablation depth, flap thickness, etc.) of other patients cannot be accurately obtained, which limits the assessment of factors for KE occurrence. In addition, age, eye rubbing, allergy, atopy, family history, and stromal hydration are related to the occurrence of KE (Randleman et al., 2003; Rabinowitz, 2006; Randleman et al., 2008; Santhiago et al., 2016). Deep research is warranted into the occurrence of KE. The present study found that the duration between corneal surgery and the onset of ectasia ranged from 4 months to 18 years, with a median time of 10 years, which is consistent with the results of several reviews (Randleman et al., 2003; Bohac et al., 2018). Males accounted for 69.6% in the current retrospective study, while a recent review reported that there was no difference between the frequency of ectasia in male and female patients (Moshirfar et al., 2021),. The basic characteristics of unilateral KE indicate the importance of follow-up of asymptomatic clinical signs, and an in-depth analysis of KE is necessary.

The corneal tomographic and biomechanical parameters were significantly different in KE vs. control eyes and KE vs. KE fellow eyes, which helped clinicians diagnose KE effectively. Among corneal tomographic parameters, PE and PRC (3 mm) are posterior evaluation parameters that have been demonstrated to be effective in differentiating KE from the control eyes. The changes in early ecstatic conditions typically occur on the posterior corneal surface before anterior changes (de Sanctis et al., 2008; Duncan et al., 2016). PRC (3 mm) is the posterior radius of curvature taken from the 3 mm zone centered on the thinnest point (Duncan et al., 2016). PE is corneal height data that uses a conventional best-fit sphere (BFS) as the reference surface (in µm), and posterior corneal elevation difference values were taken as the differential changes in corneal elevation of the thinnest points between the BFS and the enhanced BFS (de Sanctis et al., 2008; Huseynli et al., 2018). Similar to PE, FE is the elevation of the thinnest point from the 8-mm anterior corneal height data (Huseynli et al., 2018). The present study indicated that KE eyes had a higher FE value than control eyes, indicating that FE could effectively discriminate KE eyes from control eyes, and the elevation differences between a standard BFS and the enhanced reference surface were highly significant quantitatively in separating ecstatic changes from normal (Huseynli et al., 2018; Imbornoni et al., 2018; Santodomingo-Rubido et al., 2022). CBI-LVC, calculated on the basis of six dynamic corneal response parameters, had a good ability to discriminate KE from control eyes in the current analysis, which is consistent with the previous study (Vinciguerra et al., 2021). In addition, A1DLA, as a measured value in the direction parallel to the air pulse, was higher in KE eyes than that in control eyes, which is consistent with several previous studies (Ueki et al., 2018; Yang et al., 2020). The ability to distinguish KE eyes from control eyes was relatively good among the corneal biomechanical parameters, which should attract the attention of physicians for clinical applications.

According to global consensus, KC is progressive (Gomes et al., 2015). Even if one eye is not initially affected, the contralateral normal eye in most patients may eventually get affected (Degirmenci et al., 2019). Furthermore, several studies have reported that the characteristics of topographic, tomographic, and biomechanical parameters are significantly different between KC fellow eyes and normal eyes (Bae et al., 2014; Hashemi et al., 2016; Degirmenci et al., 2019). As a special type of corneal ectasia, unilateral KE was reported in the clinic; identifying differences between the KE fellow eyes and post-LASIK eyes helps detect early KE. In the present study, only PE was significantly different between KE fellow eyes and control eyes. FE and A1DLAr, which indicate the deflection area at A1, seemed to differ between the KE fellow eyes and control eyes at a wider statistical level. The results indicate that changes in the posterior cornea may occur without concurrent anterior changes, and early ectasia may have posterior progression despite a normal anterior surface (Huseynli et al., 2018; Imbornoni et al., 2018). Although the A1DLAr was not included in the final logistic regression, our previous studies found that the A1DLAr value in KE eyes was higher than that in post-LASIK control eyes, indicating that a higher value of A1DLAr suggests an increased risk of corneal ectasia (Yang et al., 2020). Further diagnostic tests showed that PE and FE had moderate AUC values in diagnosing the KE fellow eye individually, and the combination of PE and FE could effectively improve diagnostic capability. These findings further demonstrated that the differences between the elevation values, BFS, and enhanced reference surface were highly predictive in screening for ecstatic disease (Huseynli et al., 2018; Imbornoni et al., 2018). Notably, the D value was not different between the control and KE fellow eyes, which is a comprehensive display that incorporates anterior and posterior elevation, BFS, enhanced reference surface, and corneal pachymetric map into a normative database. This may be related to the variety of parameters and should be brought to the attention of clinicians. The prevalence of KE is theoretically low, although several researchers have suggested that these values are an underestimate (Santhiago et al., 2016; Bohac et al., 2018). Early diagnosis of KE is limited, especially for the KE fellow eyes of patients with unilateral KE. Overall, more attention should be paid to KE in clinical applications.

The present study showed that the combined index of PE and FE could effectively discriminate the KE fellow eye from control eyes, providing a reference for exploring the detection of corneal ectasia. However, this study has some limitations. First, the current study recruited 23 patients with unilateral KE, which is a relatively limited sample size and might fail to reflect statistical discrepancies. Considering the limited prevalence of KE and the paucity of published reports, the present study provides a reference for clinical applications and future investigations. Second, participants were recruited when they were diagnosed with ectasia at our clinic, and data on preoperative examinations and surgical procedures that might have been performed in different hospitals were lacking. More preoperative information and risk factors of KE patients need to be collected in later research. Thus, the detailed mechanism of KE has not yet been evaluated, and further studies are necessary. Finally, the study was conducted at one center, in which the ethnicity and the heterogeneity of populations were not evaluated, and extrapolation of the results requires further multicenter collaborative studies.

In conclusion, the fellow eyes of patients with unilateral KE had significantly higher PE values than control eyes, and a combination of PE and FE improved the ability to distinguish KE fellow eyes from control eyes in a Chinese population. This provides a reference for diagnosing KE early. Multicenter studies with larger sample sizes are warranted to investigate the characteristics of patients with unilateral KE and screen for early diagnostic indicators of KE.

## Data Availability

The original contributions presented in the study are included in the article/Supplementary Material; further inquiries can be directed to the corresponding author.

## Abbreviations

AUC, area under the receiver operating characteristic curve; BFS, best-fit sphere; CKI, central keratoconus index; CI, confidence interval; CBI-LVC, Corvis biomechanical index–laser vision correction; DLA, deflection amplitude; DLAr, deflection area; DA, deformation amplitude; FE, anterior evaluation; KC, keratoconus; KE, post-LASIK keratectasia; LASIK, laser-assisted *in situ* keratomileusis; and PE, posterior elevation.
