# Supplementary material for: Accuracy of tomographic and biomechanical parameters in detecting unilateral post-LASIK keratoectasia and fellow eyes
Source: Front Bioeng Biotechnol. 2023 Jun 2;11:1181117. doi: 10.3389/fbioe.2023.1181117 (PMC10272423; doi:10.3389/fbioe.2023.1181117)
Supplement: Supplementary file 1 [file Table1.DOCX]

Supplementary Material

Accuracy of tomographic and biomechanical parameters in detecting unilateral post-LASIK keratoectasia and fellow eyes

Kaili Yang^†1^, Qi Fan^†1^, Liyan Xu^1^, Yuwei Gu^1^, Chenjiu Pang^1^, Shengwei Ren^1*^

^1^ Henan Provincial People’s Hospital, Henan Eye Hospital, Henan Eye Institute, People's Hospital of Zhengzhou University, Henan University People's Hospital, Zhengzhou, 450003, China

^†^ Kaili Yang and Qi Fan have contributed equally to this work and share the first authorship

*** Correspondence:**Shengwei Ren

[shengweiren1984@163.com](mailto:shengweiren1984@163.com) and ysgzz2018@163.com

# Supplementary Tables

**1.1 Supplemental Table 1** Comparison of corneal tomographic parameters among control, KE and the fellow eyes, M(P25, P75).

**1.2 Supplemental Table 2** Comparison of the biomechanical parameters among control, KE and the fellow eyes, M(P25, P75).

**1.3 Supplemental Table 3** Ability of corneal tomographic parameters in distinguishing KE eye from control eyes.

**1.4 Supplemental Table 4** Ability of corneal biomechanical parameters in distinguishing KE eye from control eyes.

**1.5 Supplemental Table 5** Logistic regression for predicting KE fellow eye

**1.6 Supplemental Table 6** Preoperative information of KE patients

# Supplementary Figure

**2.1** **Supplemental Figure 1** The flap thickness measurements of 23 patients. N1-N4 and N6-N23 patient was measured through CASIA SS-1000, and N5 patient was measured through Visante OCT (Zeiss).

**Supplemental Table 1** Comparison of corneal tomographic parameters among control, KE and the fellow eyes.

| **Parameters** | **Control group**  **(n=48)** | |  | **Fellow eye of KE**  **(n=23)** | |  | **KE group**  **(n=23)** | | **P*** | **P#1** | **P#2** | **P#3** |
| --- | --- | --- | --- | --- | --- | --- | --- | --- | --- | --- | --- | --- |
|  | **mean±SD** | **M(P25, P75)** |  | **mean±SD** | **M(P25, P75)** |  | **mean±SD** | **M(P25, P75)** |  |  |  |  |
| K1F(D) | 38.35±2.48 | 38.85(36.43, 39.98) |  | 39.5±2.07 | 39.7(37.8, 40.9) |  | 44.23±4.39 | 43.7(40.5, 47.3) | <0.001 | 0.316 | <0.001 | 0.003 |
| K2F(D) | 39.33±2.7 | 39.8(36.95, 41.5) |  | 40.47±2.24 | 40.9(38.7, 41.7) |  | 47.05±5.01 | 45.3(42.8, 51.6) | <0.001 | 0.492 | <0.001 | <0.001 |
| KmF(D) | 38.84±2.56 | 39.4(36.73, 40.7) |  | 39.99±2.14 | 40.3(38.3, 41.3) |  | 45.57±4.58 | 44.5(41.5, 49.2) | <0.001 | 0.372 | <0.001 | <0.001 |
| KMax(D) | 44.64±2.17 | 44.11(43, 45.77) |  | 45.3±5.32 | 44.3(43.2, 45.2) |  | 52.87±6.56 | 52.4(47.8, 56.6) | <0.001 | 1.000 | <0.001 | <0.001 |
| ACT | 472.98±47.02 | 475.5(426.5, 511) |  | 474.87±36.64 | 477(448, 496) |  | 432±37.07 | 439(388, 461) | 0.002 | 1.000 | 0.003 | 0.006 |
| PCT | 470.17±48.1 | 471(420.25, 509.75) |  | 474.83±36.47 | 473(449, 494) |  | 439.7±40.2 | 449(400, 464) | 0.016 | 1.000 | 0.037 | 0.028 |
| TCT | 468.31±48.29 | 469.5(419, 509.75) |  | 472.17±36.91 | 470(445, 491) |  | 427.26±37.8 | 436(385, 448) | 0.002 | 1.000 | 0.004 | 0.005 |
| ARC(3mmZone) | 8.8±0.63 | 8.69(8.25, 9.3) |  | 8.44±0.44 | 8.35(8.07, 8.84) |  | 7.18±0.77 | 7.15(6.63, 7.71) | <0.001 | 0.190 | <0.001 | <0.001 |
| PRC(3mmZone) | 6.49±0.22 | 6.51(6.32, 6.67) |  | 6.36±0.27 | 6.32(6.09, 6.55) |  | 4.94±0.63 | 4.94(4.27, 5.56) | <0.001 | 0.418 | <0.001 | <0.001 |
| ISV | 42.92±21.89 | 41(25.25, 55.75) |  | 32.26±6.52 | 32(29, 37) |  | 77.04±32.96 | 79(48, 100) | <0.001 | 0.288 | <0.001 | <0.001 |
| IVA | 0.43±0.29 | 0.39(0.18, 0.56) |  | 0.31±0.09 | 0.29(0.25, 0.39) |  | 0.85±0.43 | 0.82(0.51, 1.16) | <0.001 | 0.579 | <0.001 | <0.001 |
| KI | 1±0.07 | 1(0.94, 1.03) |  | 0.99±0.05 | 1(0.96, 1.02) |  | 1.16±0.12 | 1.16(1.08, 1.25) | <0.001 | 1.000 | <0.001 | <0.001 |
| CKI | 0.98±0.02 | 0.99(0.97, 1) |  | 0.99±0.01 | 0.99(0.98, 1) |  | 1.07±0.06 | 1.06(1.02, 1.1) | <0.001 | 0.206 | <0.001 | <0.001 |
| IHA | 11.49±10.59 | 10.35(2.88, 15.08) |  | 14.25±7.86 | 11.4(7.8, 19) |  | 23.8±24.59 | 11(6, 37.2) | 0.075 |  |  |  |
| IHD | 0.04±0.03 | 0.03(0.02, 0.05) |  | 0.03±0.01 | 0.03(0.02, 0.04) |  | 0.1±0.06 | 0.11(0.05, 0.15) | <0.001 | 1.000 | <0.001 | <0.001 |
| **FE** | -7.38±7.38 | **-6(-9.75, -2.25)** |  | -2.74±2.7 | **-3(-4, -2)** |  | 14.17±9.71 | **15(5, 19)** | **<0.001** | **0.062** | **<0.001** | **<0.001** |
| **PE** | 2.44±3.11 | **2(0, 4.75)** |  | 5.17±2.82 | **5(3, 7)** |  | 48.17±23.32 | **46(25, 68)** | **<0.001** | **0.035** | **<0.001** | **<0.001** |
| PPImin | 1.6±0.5 | 1.49(1.21, 1.91) |  | 1.41±0.32 | 1.39(1.2, 1.65) |  | 2.5±0.79 | 2.44(1.9, 2.96) | <0.001 | 0.842 | <0.001 | <0.001 |
| PPImax | 2.37±0.65 | 2.3(1.88, 2.68) |  | 2.08±0.45 | 2.03(1.83, 2.37) |  | 3.99±1.04 | 3.96(3.16, 4.56) | <0.001 | 0.429 | <0.001 | <0.001 |
| PPImean | 1.98±0.55 | 1.93(1.54, 2.29) |  | 1.73±0.35 | 1.72(1.49, 1.95) |  | 2.98±0.76 | 2.85(2.44, 3.47) | <0.001 | 0.422 | <0.001 | <0.001 |
| D | 3.69±1.82 | 3.54(2.19, 4.84) |  | 2.99±0.9 | 3.12(2.19, 3.67) |  | 9.69±5.48 | 8.11(5.51, 11.73) | <0.001 | 0.602 | <0.001 | <0.001 |

K1F, flat keratometry front; K2F, steep keratometry front; KmF, mean keratometry front; Kmax, the maximum keratometry; ACT, corneal thickness at the pachy apex; PCT, corneal thickness at the pupil's center; TCT, corneal thickness at the thinnest point of the cornea; FE, Height of the thinnest corneal point on the anterior surface of the cornea; PE, Height of the thinnest corneal point on the posterior surface of the cornea; ISV, index of surface variation; IVA, index of vertical asymmetry; KI, keratoconus index; CKI, central keratoconus index; IHA, index of height asymmetry; IHD, index height decentration; PPI, pachymetric progression index; D, Belin-Ambrosio Deviation.

*, Kruskal-Wallis test of three groups; #1, comparison between fellow eye of KE and control eye; #2, comparison between KE and control eye; #3, comparison between KE and fellow eye.

**Supplemental Table 2** Comparison of the biomechanical parameters among control, KE and the fellow eyes.

| **Parameters** | **Control group**  **(n=48)** | |  | **Fellow eye of KE**  **(n=23)** | |  | **KE group**  **(n=23)** | | **P*** | **P#1** | **P#2** | **P#3** |
| --- | --- | --- | --- | --- | --- | --- | --- | --- | --- | --- | --- | --- |
|  | **mean±SD** | **M(P25, P75)** |  | **mean±SD** | **M(P25, P75)** |  | **mean±SD** | **M(P25, P75)** |  |  |  |  |
| IOP(mmHg) | 13±2.18 | 13.25(11.63, 14.38) |  | 12.78±2.12 | 12.5(12, 14.5) |  | 12.46±2.59 | 12.5(11, 14) | 0.573 |  |  |  |
| CCT(µm) | 462.09±47.51 | 462(418.5, 499.5) |  | 471.78±38.82 | 469(444, 496) |  | 439.7±41.29 | 454(403, 471) | 0.076 |  |  |  |
| DAMax(mm) | 1.15±0.09 | 1.14(1.08, 1.19) |  | 1.15±0.13 | 1.1(1.06, 1.23) |  | 1.25±0.12 | 1.2(1.17, 1.36) | 0.001 | 1.000 | 0.002 | 0.005 |
| A1T(ms) | 6.85±0.26 | 6.82(6.68, 7.02) |  | 6.94±0.36 | 6.89(6.67, 7.23) |  | 6.91±0.39 | 6.8(6.6, 7.22) | 0.574 |  |  |  |
| A1V(m/s) | 0.15±0.02 | 0.15(0.14, 0.15) |  | 0.15±0.02 | 0.14(0.13, 0.15) |  | 0.16±0.02 | 0.16(0.15, 0.18) | 0.005 | 1.000 | 0.006 | 0.027 |
| A2T(ms) | 22.34±0.38 | 22.35(22.17, 22.54) |  | 22.31±0.34 | 22.17(22.04, 22.53) |  | 22.41±0.5 | 22.38(22.1, 22.62) | 0.615 |  |  |  |
| A2V(m/s) | -0.27±0.04 | -0.27(-0.3, -0.25) |  | -0.29±0.05 | -0.28(-0.3, -0.25) |  | -0.32±0.05 | -0.32(-0.35, -0.29) | 0.001 | 1.000 | 0.001 | 0.023 |
| HCT(ms) | 17.52±0.54 | 17.56(17.33, 17.79) |  | 17.43±0.7 | 17.56(16.86, 17.79) |  | 17.45±0.68 | 17.56(17.09, 17.79) | 0.784 |  |  |  |
| PD(mm) | 5.31±0.21 | 5.3(5.15, 5.47) |  | 5.21±0.37 | 5.21(4.86, 5.49) |  | 5.2±0.29 | 5.17(4.96, 5.46) | 0.243 |  |  |  |
| Radius(mm) | 5.85±0.61 | 5.76(5.5, 6.1) |  | 5.6±0.6 | 5.61(5.15, 5.89) |  | 4.88±0.79 | 5.14(4.38, 5.35) | <0.001 | 0.376 | <0.001 | 0.022 |
| A1DA(mm) | 0.11±0.01 | 0.11(0.1, 0.12) |  | 0.11±0.01 | 0.11(0.1, 0.12) |  | 0.13±0.01 | 0.13(0.12, 0.14) | <0.001 | 1.000 | <0.001 | <0.001 |
| HCDA(mm) | 1.15±0.09 | 1.14(1.08, 1.19) |  | 1.15±0.13 | 1.1(1.06, 1.23) |  | 1.25±0.12 | 1.2(1.17, 1.36) | 0.001 | 1.000 | 0.002 | 0.005 |
| A2DA(mm) | 0.35±0.06 | 0.35(0.3, 0.39) |  | 0.35±0.07 | 0.36(0.29, 0.42) |  | 0.38±0.07 | 0.37(0.32, 0.43) | 0.244 |  |  |  |
| A1DLL(mm) | 1.88±0.22 | 1.82(1.72, 2.04) |  | 2±0.21 | 2.02(1.84, 2.09) |  | 2.13±0.27 | 2.13(2, 2.24) | 0.001 | 0.141 | 0.001 | 0.364 |
| HCDLL(mm) | 6.61±0.39 | 6.62(6.37, 6.88) |  | 6.51±0.57 | 6.49(6.13, 6.85) |  | 6.29±0.53 | 6.35(5.87, 6.71) | 0.083 |  |  |  |
| A2DLL(mm) | 2.7±1 | 2.64(1.78, 3.64) |  | 2.88±0.95 | 3.02(2.13, 3.84) |  | 2.53±0.9 | 2.31(1.85, 2.9) | 0.427 |  |  |  |
| A1DLA(mm) | 0.07±0.01 | 0.07(0.07, 0.08) |  | 0.08±0.01 | 0.08(0.07, 0.09) |  | 0.1±0.01 | 0.1(0.09, 0.11) | <0.001 | 0.164 | <0.001 | <0.001 |
| HCDLA(mm) | 0.99±0.09 | 0.99(0.92, 1.05) |  | 1±0.13 | 0.98(0.92, 1.09) |  | 1.09±0.13 | 1.06(1, 1.17) | 0.007 | 1.000 | 0.009 | 0.026 |
| A2DLA(mm) | 0.08±0.01 | 0.08(0.07, 0.09) |  | 0.09±0.04 | 0.08(0.08, 0.1) |  | 0.11±0.02 | 0.1(0.1, 0.12) | <0.001 | 0.308 | <0.001 | 0.001 |
| DLAML(mm) | 1±0.09 | 0.99(0.94, 1.06) |  | 1.02±0.12 | 0.98(0.93, 1.11) |  | 1.11±0.12 | 1.08(1, 1.2) | 0.002 | 1.000 | 0.003 | 0.017 |
| DLAMT(ms) | 16.4±0.69 | 16.6(15.75, 16.98) |  | 16.36±0.82 | 16.39(15.82, 16.88) |  | 16.35±0.89 | 16.55(15.76, 17.03) | 0.906 |  |  |  |
| WEMA(mm) | 0.28±0.06 | 0.28(0.23, 0.32) |  | 0.27±0.08 | 0.26(0.22, 0.33) |  | 0.28±0.07 | 0.27(0.22, 0.32) | 0.850 |  |  |  |
| WEMT(ms) | 22.02±0.55 | 22.03(21.67, 22.24) |  | 22.11±0.59 | 21.96(21.71, 22.37) |  | 22.27±0.82 | 22.07(21.81, 22.41) | 0.529 |  |  |  |
| **A1DLAr(mm^2^)** | 0.12±0.03 | **0.12(0.1, 0.15)** |  | 0.14±0.03 | **0.15(0.12, 0.16)** |  | 0.16±0.04 | **0.16(0.14, 0.18)** | **<0.001** | **0.073** | **<0.001** | 0.340 |
| HCDLAr(mm^2^) | 3.61±0.49 | 3.61(3.26, 3.94) |  | 3.57±0.74 | 3.48(3.2, 4.04) |  | 3.77±0.6 | 3.61(3.31, 4.29) | 0.459 |  |  |  |
| A2DLAr(mm^2^) | 0.15±0.04 | 0.14(0.12, 0.18) |  | 0.19±0.1 | 0.17(0.13, 0.2) |  | 0.21±0.07 | 0.2(0.17, 0.27) | 0.001 | 0.187 | <0.001 | 0.263 |
| A1dArcL(mm) | -0.01±0 | -0.01(-0.01, -0.01) |  | -0.01±0 | -0.01(-0.01, -0.01) |  | -0.01±0.01 | -0.01(-0.02, -0.01) | <0.001 | 0.177 | <0.001 | 0.174 |
| HCdArcL(mm) | -0.07±0.03 | -0.07(-0.1, -0.05) |  | -0.08±0.03 | -0.07(-0.1, -0.05) |  | -0.08±0.04 | -0.07(-0.1, -0.04) | 0.684 |  |  |  |
| A2dArcL(mm) | -0.01±0.01 | -0.01(-0.01, -0.01) |  | -0.01±0.01 | -0.01(-0.02, -0.01) |  | -0.02±0.01 | -0.02(-0.03, -0.01) | 0.004 | 0.162 | 0.005 | 0.840 |
| dArcLM(mm) | -0.09±0.03 | -0.09(-0.11, -0.06) |  | -0.1±0.03 | -0.1(-0.12, -0.08) |  | -0.1±0.04 | -0.09(-0.12, -0.06) | 0.363 |  |  |  |
| Max Inverse Radius(mm^-1^) | 0.21±0.02 | 0.21(0.2, 0.22) |  | 0.21±0.02 | 0.22(0.2, 0.23) |  | 0.26±0.04 | 0.25(0.23, 0.29) | <0.001 | 1.000 | <0.001 | <0.001 |
| DA Ratio Max[2mm] | 5.5±0.51 | 5.49(5.07, 5.92) |  | 5.59±0.53 | 5.62(5.19, 6.03) |  | 6.93±1.53 | 6.44(5.75, 7.76) | <0.001 | 1.000 | <0.001 | 0.001 |
| Pachy Slope(µm) | 90.43±20.49 | 88.53(77.42, 100.03) |  | 81.03±17.76 | 83.45(71.52, 94.07) |  | 118.78±34.48 | 111.13(92.6, 142.6) | <0.001 | 0.520 | 0.002 | <0.001 |
| DA Ratio Max[1mm] | 1.71±0.05 | 1.72(1.68, 1.74) |  | 1.72±0.05 | 1.73(1.68, 1.76) |  | 1.8±0.09 | 1.79(1.74, 1.84) | <0.001 | 1.000 | <0.001 | 0.006 |
| ARTh | 188.98±71.76 | 169.81(132.86, 248.92) |  | 231.89±111.4 | 205.24(165.05, 261.13) |  | 115.7±43.38 | 119.49(68.9, 148.93) | <0.001 | 0.245 | <0.001 | <0.001 |
| bIOP(mmHg) | 14.66±1.85 | 14.45(13.6, 15.88) |  | 14.39±1.58 | 14.4(13.5, 15.8) |  | 14.6±2.71 | 14.3(12.6, 16.4) | 0.785 |  |  |  |
| Integrated Radius(mm^-1^) | 11.28±1.01 | 11.23(10.68, 11.89) |  | 11.4±1.07 | 11.25(10.44, 12.4) |  | 13.82±2.74 | 13.15(12.26, 15.2) | <0.001 | 1.000 | <0.001 | 0.001 |
| SP-A1(mmHg/mm) | 94.65±23.4 | 93.86(80.06, 107.92) |  | 86.58±19.35 | 85.53(77.83, 100.93) |  | 62.8±15.36 | 60.63(56.93, 72.73) | <0.001 | 0.719 | <0.001 | 0.001 |
| CBI-LVC | 0.14±0.28 | 0(0, 0.08) |  | 0.42±0.43 | 0.29(0, 0.92) |  | 0.96±0.21 | 1(1, 1) | <0.001 | 0.102 | <0.001 | 0.009 |

IOP, intraocular pressure; CCT, central corneal thickness; DA, deformation amplitude; A1, the first applanation; HC, the highest concavity; A2, the second applanation; PD, peak distance; DLL, deflection length; DLA, deflection amplitude; DLAr, deflection area; dArcL, delta arc length; WEMA, amplitude of whole eye movement; WEMT, time of whole eye movement; ARTh, Ambrósio’s relational thickness horizontal; SP-A1, stiffness parameter at first applanation; bIOP, biomechanical corrected IOP; CBI-LVC, Corvis biomechanical index–laser vision correction.

*, Kruskal-Wallis test of three groups; #1, comparison between fellow eye of KE and control eye; #2, comparison between KE and control eye; #3, comparison between KE and fellow eye.

**Supplemental Table 3** Ability of corneal tomographic parameters in distinguishing KE eye from control eyes.

| **Parameters** | **Cut off** | **AUC(95%CI)** | **Youden index** | **Sensitivity(%)** | **Specificity(%)** |
| --- | --- | --- | --- | --- | --- |
| K1F(D) | >41.4 | 0.882(0.784, 0.947) | 0.635 | 73.91 | 89.58 |
| K2F(D) | >41.8 | 0.933(0.848, 0.979) | 0.767 | 91.30 | 85.42 |
| KmF(D) | >40.8 | 0.917(0.827, 0.969) | 0.726 | 91.30 | 81.25 |
| KMax(D) | >45.78 | 0.893(0.791, 0.956) | 0.699 | 91.30 | 78.57 |
| ACT | ≤464 | 0.731(0.613, 0.829) | 0.430 | 82.61 | 60.42 |
| PCT | ≤478 | 0.676(0.554, 0.782) | 0.328 | 86.96 | 45.83 |
| TCT | ≤475 | 0.725(0.606, 0.824) | 0.415 | 95.65 | 45.83 |
| ARC(3mmZone) | ≤8.06 | 0.943(0.862, 0.984) | 0.788 | 91.30 | 87.50 |
| **PRC(3mmZone)** | **≤5.94** | **1(0.949, 1.000)** | **1.000** | **100.00** | **100.00** |
| ISV | >62 | 0.808(0.698, 0.892) | 0.506 | 65.22 | 85.42 |
| IVA | >0.61 | 0.813(0.703, 0.896) | 0.529 | 69.57 | 83.33 |
| KI | >1.03 | 0.915(0.824, 0.968) | 0.767 | 91.30 | 85.42 |
| CKI | >1 | 0.988(0.928, 1.000) | 0.915 | 95.65 | 95.83 |
| IHA | >17.4 | 0.634(0.511, 0.745) | 0.353 | 47.83 | 87.50 |
| IHD | >0.058 | 0.856(0.753, 0.928) | 0.614 | 73.91 | 87.50 |
| **FE** | **>-1** | **0.996(0.942, 1.000)** | **0.938** | **100.00** | **93.75** |
| **PE** | **>9** | **1(0.949, 1.000)** | **1.000** | **100.00** | **100.00** |
| PPImin | >1.8 | 0.848(0.743, 0.922) | 0.578 | 86.96 | 70.83 |
| PPImax | >2.86 | 0.921(0.832, 0.972) | 0.705 | 91.30 | 79.17 |
| PPImean | >2.31 | 0.862(0.760, 0.933) | 0.597 | 82.61 | 77.08 |
| D | >4.81 | 0.924(0.836, 0.973) | 0.686 | 95.65 | 72.92 |

K1F, flat keratometry front; K2F, steep keratometry front; KmF, mean keratometry front; Kmax, the maximum keratometry; ACT, corneal thickness at the pachy apex; PCT, corneal thickness at the pupil's center; TCT, corneal thickness at the thinnest point of the cornea; FE, Height of the thinnest corneal point on the anterior surface of the cornea; PE, Height of the thinnest corneal point on the posterior surface of the cornea; ISV, index of surface variation; IVA, index of vertical asymmetry; KI, keratoconus index; CKI, central keratoconus index; IHA, index of height asymmetry; IHD, index height decentration; PPI, pachymetric progression index; D, Belin-Ambrosio Deviation.

**Supplemental Table 4** Ability of corneal biomechanical parameters in distinguishing KE eye from control eyes.

| **Parameters** | **Cut off** | **AUC(95%CI)** | **Youden**  **index** | **Sensitivity**  **(%)** | **Specificity**  **(%)** |
| --- | --- | --- | --- | --- | --- |
| DA Max(mm) | >1.179 | 0.77(0.651, 0.864) | 0.466 | 73.91 | 72.73 |
| A1V(m/s) | >0.156 | 0.732(0.609, 0.833) | 0.470 | 65.22 | 81.82 |
| A2V(m/s) | ≤-0.319 | 0.773(0.655, 0.867) | 0.431 | 52.17 | 90.91 |
| Radius(mm) | ≤5.412 | 0.838(0.728, 0.917) | 0.622 | 82.61 | 79.55 |
| A1DA(mm) | >0.118 | 0.934(0.845, 0.980) | 0.756 | 86.96 | 88.64 |
| HCDA(mm) | >1.179 | 0.77(0.651, 0.864) | 0.466 | 73.91 | 72.73 |
| A1DLL(mm) | >1.934 | 0.765(0.645, 0.860) | 0.485 | 82.61 | 65.91 |
| **A1DLA(mm)** | **>0.084** | **0.952(0.870, 0.989)** | **0.797** | **95.65** | **84.09** |
| HCDLA(mm) | >1.033 | 0.731(0.609, 0.832) | 0.379 | 65.22 | 72.73 |
| A2DLA(mm) | >0.098 | 0.912(0.817, 0.967) | 0.671 | 73.91 | 93.18 |
| DLAML(mm) | >0.968 | 0.752(0.632, 0.850) | 0.388 | 95.65 | 43.18 |
| A1DLAr(mm^2^) | >0.155 | 0.785(0.668, 0.876) | 0.493 | 65.22 | 84.09 |
| A2DLAr(mm^2^) | >0.16 | 0.776(0.657, 0.869) | 0.464 | 78.26 | 68.18 |
| A1dArcL(mm) | ≤-0.012 | 0.784(0.666, 0.875) | 0.576 | 82.61 | 75.00 |
| A2dArcL(mm) | ≤-0.016 | 0.732(0.610, 0.833) | 0.406 | 56.52 | 84.09 |
| Max Inverse Radius(mm^-1^) | >0.222 | 0.89(0.790, 0.954) | 0.686 | 91.30 | 77.27 |
| DA Ratio Max[2mm] | >6.076 | 0.85(0.741, 0.925) | 0.605 | 69.57 | 90.91 |
| Pachy Slope(µm) | >103.626 | 0.764(0.644, 0.859) | 0.493 | 65.22 | 84.09 |
| DA Ratio Max[1mm] | >1.763 | 0.813(0.699, 0.898) | 0.582 | 69.57 | 88.64 |
| ARTh | ≤122.965 | 0.795(0.679, 0.884) | 0.427 | 60.87 | 81.82 |
| Integrated Radius(mm^-1^) | >11.954 | 0.838(0.728, 0.917) | 0.622 | 82.61 | 79.55 |
| SP-A1(mmHg/mm) | ≤76.595 | 0.892(0.793, 0.955) | 0.711 | 86.96 | 84.09 |
| **CBI-LVC** | **>0.918** | **0.983(0.917-0.999)** | **0.957** | **95.65** | **100** |

DA, deformation amplitude; A1, the first applanation; HC, the highest concavity; A2, the second applanation; PD, peak distance; DLL, deflection length; DLA, deflection amplitude; DLAr, deflection area; dArcL, delta arc length; WEMA, amplitude of whole eye movement; WEMT, time of whole eye movement; ARTh, Ambrósio’s relational thickness horizontal; SP-A1, stiffness parameter at first applanation; CBI-LVC, Corvis biomechanical index–laser vision correction–laser vision correction.

**Supplemental Table 5** Logistic regression for predicting KE fellow eye

| **Parameters** | **B** | **SE** | ***P*** | **OR** | **95%CI** |
| --- | --- | --- | --- | --- | --- |
| FE | 0.264 | 0.109 | 0.015 | 1.302 | 1.053-1.611 |
| PE | 0.343 | 0.114 | 0.003 | 1.410 | 1.127-1.763 |
| Constant | -0.716 | 0.642 | 0.265 | 0.489 | - |

FE, Height of the thinnest corneal point on the anterior surface of the cornea; PE, Height of the thinnest corneal point on the posterior surface.

**Supplemental Table 6** Preoperative information of KE patients

| **NO** | **KE eye** | **Preoperative Refraction(OD)** | **Preoperative Refraction(OS)** | **Preoperative Pachymetry(OD)** | **Preoperative Pachymetry(OS)** |
| --- | --- | --- | --- | --- | --- |
| N7 | Right | -4.25+1.00×15 | -3.75+0.75×155 | 496 | 494 |
| N9 | Left | -6.25+1.00×5 | -6.50+0.75×150 | 543 | 529 |
| N16 | Left | -5.75+0.50×5 | -5.75+1.25×170 | 515 | 525 |
| N20 | Right | -6.00+0.50×70 | -6.00+1.00×175 | 518 | 526 |
| N21 | Left | -6.75+0.50×70 | -7.00+0.50×145 | 509 | 505 |
