# Supplementary material for: Accuracy of tomographic and biomechanical parameters in detecting unilateral post-LASIK keratoectasia and fellow eyes
Source: Front Bioeng Biotechnol. 2023 Jun 2;11:1181117. doi: 10.3389/fbioe.2023.1181117 (PMC10272423; doi:10.3389/fbioe.2023.1181117)
Supplement: Supplementary file 2 [file DataSheet1.PDF]

N1

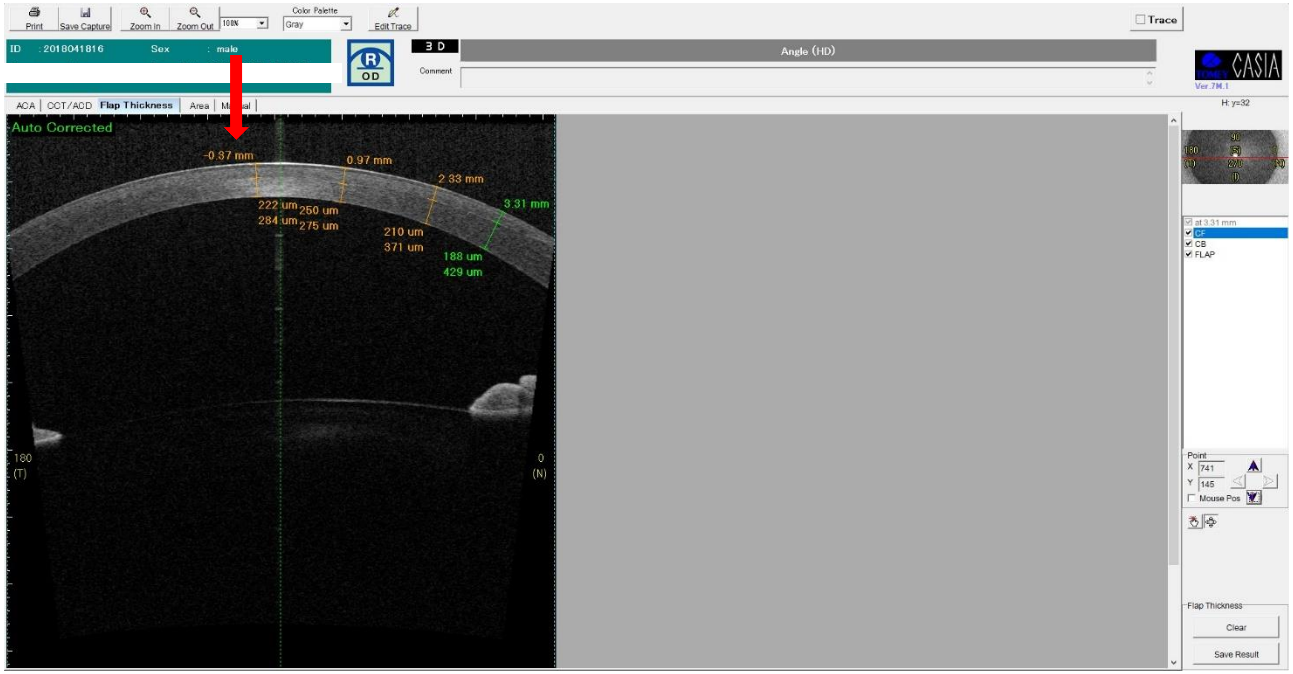
Print
Save Capture
Zoom In
Zoom Out
Color Palette
Gray
Edit Trace
Trace
ID : 2018041816
Sex : male
OD
3 D
Angle (HD)
Comment
CASIA
ACA
CCT/ACD
Flap Thickness
Area
Auto Corrected
-0.37 mm
0.97 mm
2.33 mm
3.31 mm
222 um
284 um
250 um
275 um
210 um
371 um
188 um
429 um
180
(T)
0
(N)
CF
CB
FLAP
Point
Mouse Pos
Clear
Save Result

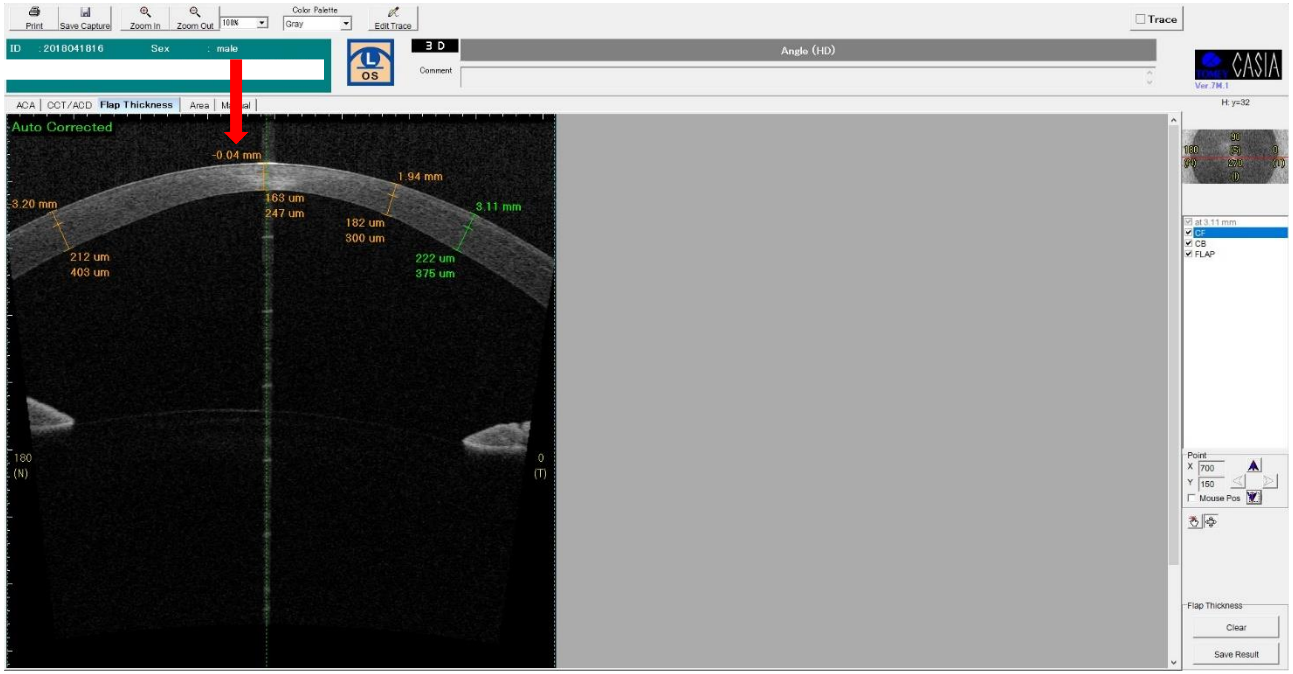
Print
Save Capture
Zoom In
Zoom Out
Color Palette
Gray
Edit Trace
Trace
ID : 2018041816
Sex : male
OS
3 D
Angle (HD)
Comment
CASIA
ACA
CCT/ACD
Flap Thickness
Area
Auto Corrected
-0.04 mm
1.94 mm
3.11 mm
3.20 mm
163 um
247 um
182 um
300 um
212 um
403 um
222 um
375 um
180
(N)
0
(T)
CF
CB
FLAP
Point
Mouse Pos
Clear
Save Result

N2

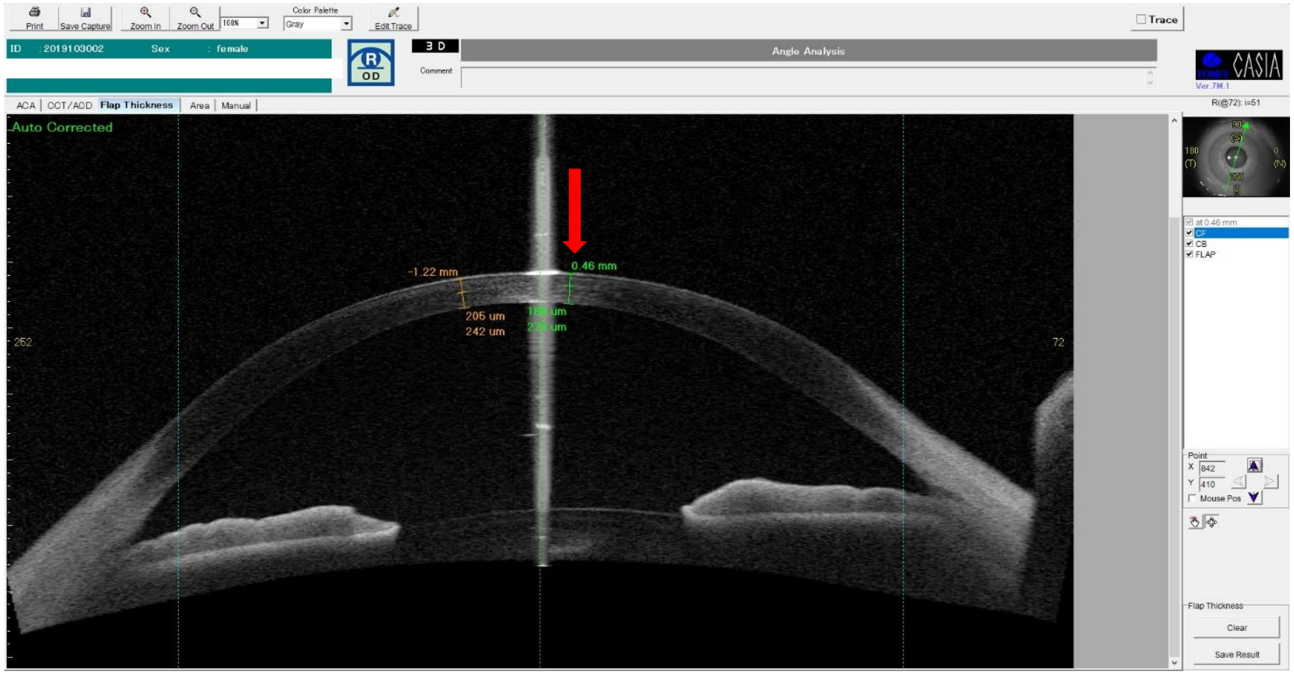

Print
Save Capture
Zoom In
Zoom Out
Color Palette
Gray
Edit Trace
Trace
ID : 2019103002
Sex : female
R OD
3 D
Angle Analysis
Comment
CASIA
ACA
OCT/ACD
Flap Thickness
Area
Manual
Auto Corrected
-1.22 mm
0.46 mm
205 um
242 um
252
72
Point
Mouse Pos
Clear
Save Result

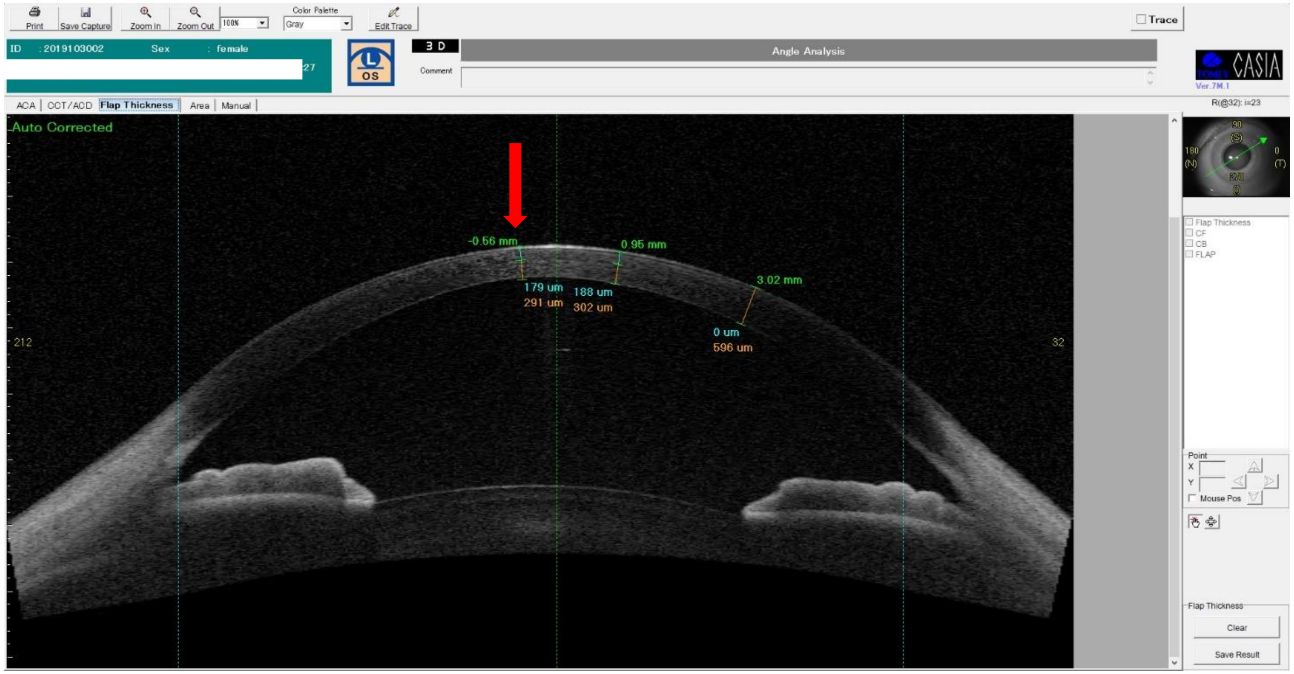

Print
Save Capture
Zoom In
Zoom Out
Color Palette
Gray
Edit Trace
Trace
ID : 2019103002
Sex : female
L OS
3 D
Angle Analysis
Comment
CASIA
ACA
OCT/ACD
Flap Thickness
Area
Manual
Auto Corrected
-0.56 mm
0.95 mm
3.02 mm
179 um
291 um
188 um
302 um
0 um
596 um
212
32
Flap Thickness
CF
CB
FLAP
Point
Mouse Pos
Clear
Save Result

N3

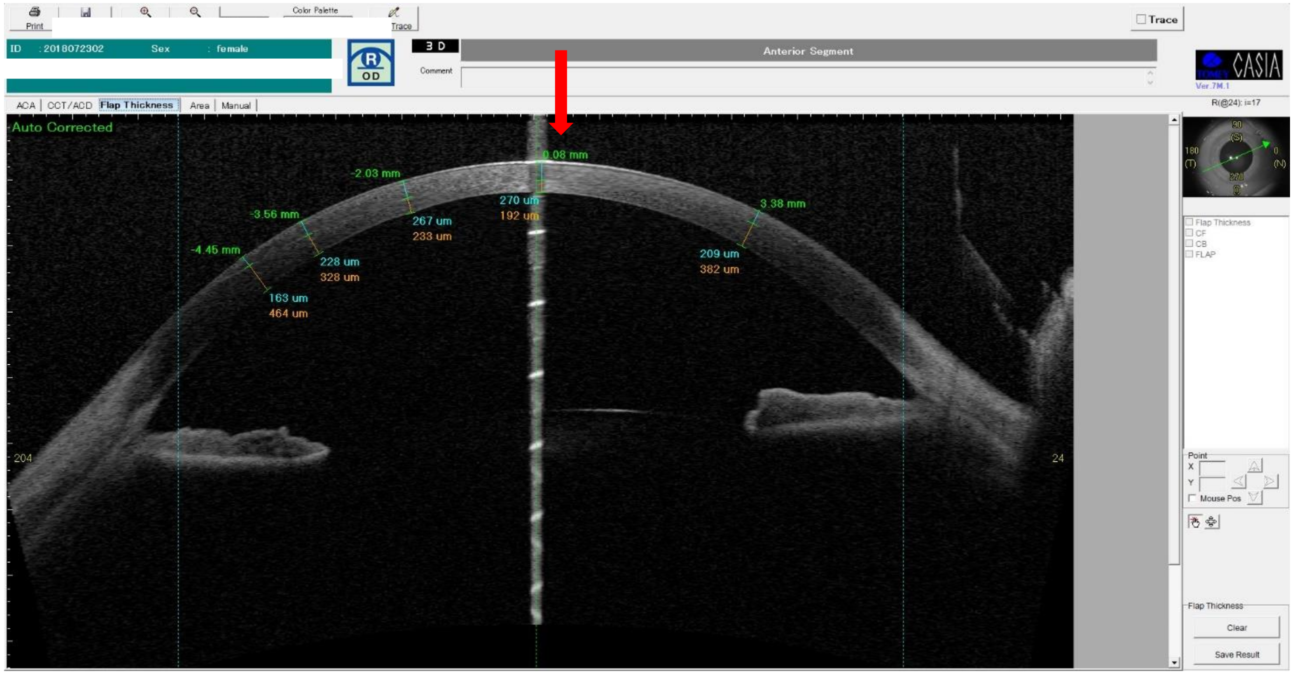

Print
Color Palette
Trace
ID : 2018072302
Sex : female
R
OD
3 D
Anterior Segment
Comment
CASIA
ACA
CCT/ACD
Flap Thickness
Area
Manual
Auto Corrected
0.08 mm
-2.03 mm
-3.56 mm
-4.45 mm
3.38 mm
270 um
192 um
267 um
233 um
228 um
328 um
163 um
464 um
209 um
382 um
204
24
Flap Thickness
CF
CB
FLAP
Point
Mouse Pos
Clear
Save Result

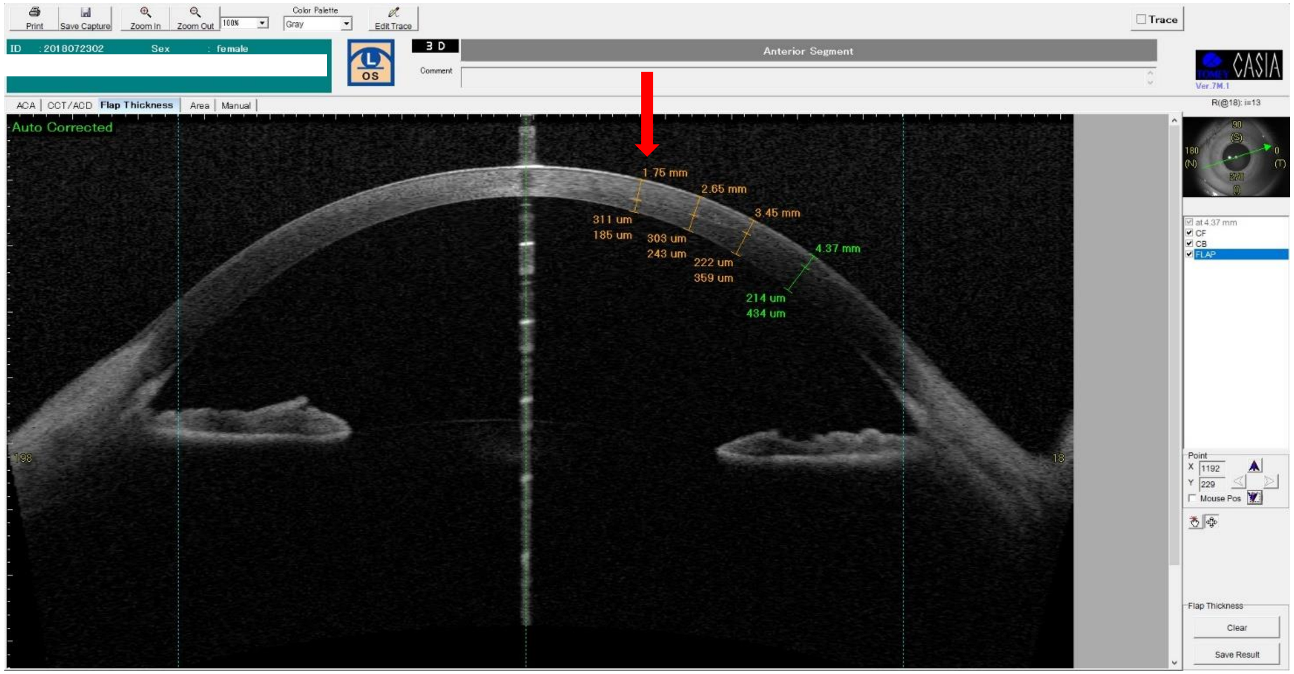

Print
Save Capture
Zoom In
Zoom Out
Color Palette
Gray
Edit Trace
Trace
ID : 2018072302
Sex : female
L
OS
3 D
Anterior Segment
Comment
CASIA
ACA
CCT/ACD
Flap Thickness
Area
Manual
Auto Corrected
1.75 mm
2.65 mm
3.45 mm
4.37 mm
311 um
185 um
303 um
243 um
222 um
359 um
214 um
434 um
CF
CB
FLAP
Point
1192
229
Mouse Pos
Clear
Save Result

N4

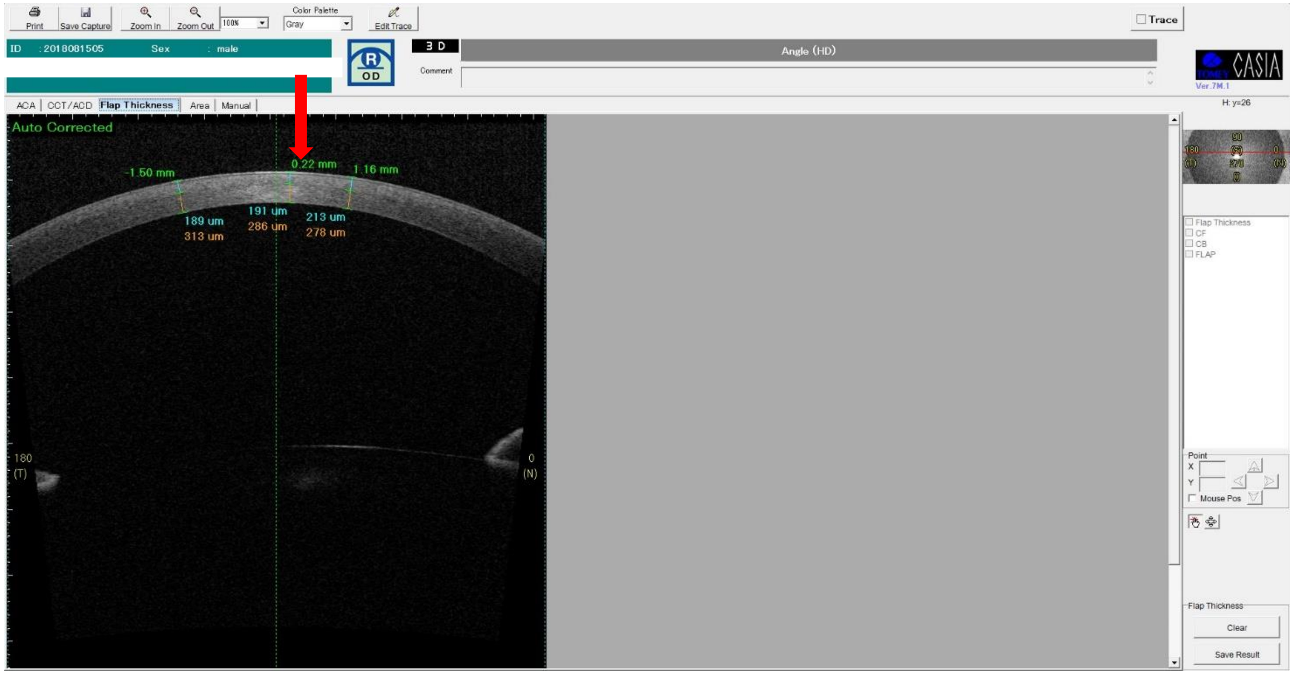
Auto Corrected
-1.50 mm
0.22 mm
1.16 mm
189 um
313 um
191 um
286 um
213 um
278 um
180
(T)
0
(N)
Angle (HD)
OD
Flap Thickness

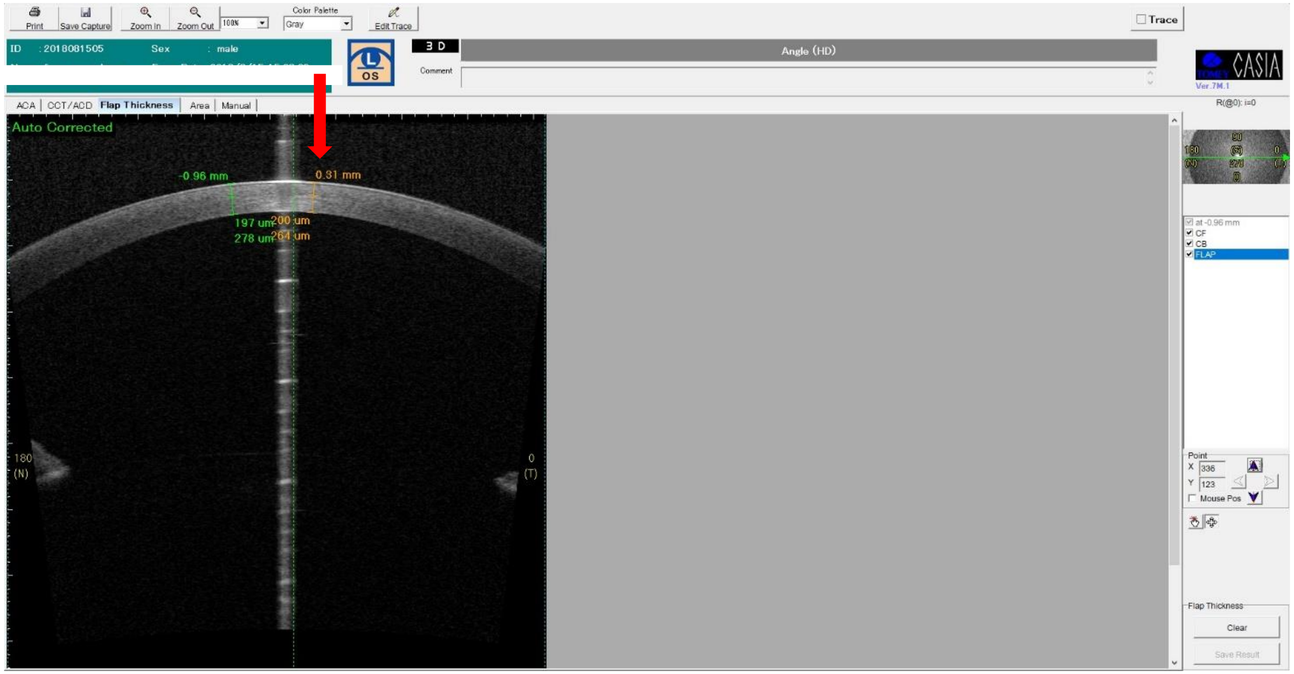
Auto Corrected
-0.96 mm
0.31 mm
197 um
278 um
200 um
264 um
180
(N)
0
(T)
Angle (HD)
OS
Flap Thickness

N5

Image Analysis Report

ZEISS

OD

Visante™ OCT
ANTERIOR SEGMENT IMAGING

Patient name: j

Date of birth: 9/12/1983
Patient ID: A15757

Exam date: 10/15/2018 11:36:42 AM

Rx: Sphere: 0.0
Cylinder: 0.0
Axis: 0.0
Fixation Angle: 0.0
Polarization: 33.0
Orientation: 0.0

Protocol: LVC Pachymetry And Topography
Scan: Enhanced High Res. Corneal

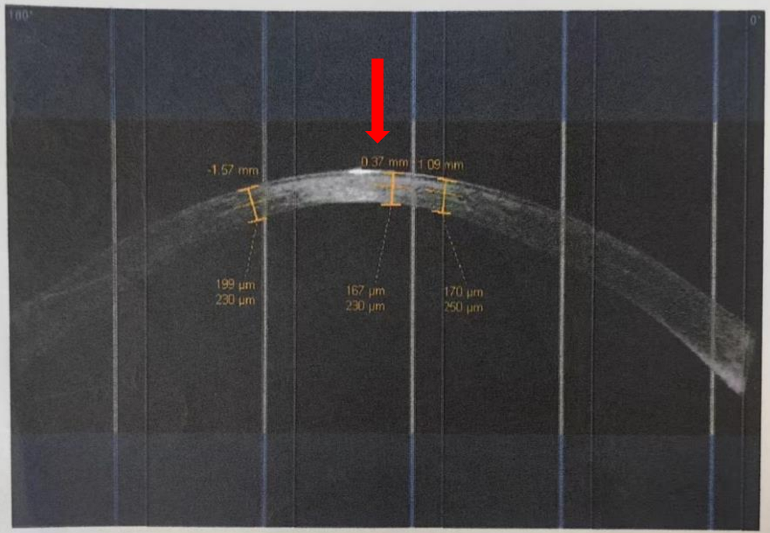

Planning

Institution:
Planner: operator

Date: 10/15/2018 11:39:47 AM Serial Number: 1000-2193
Station: henan province people's Software Ver: 3.0.1.8

Acquisition

Institution:
Operator: operator

Serial Number: 1000-2193
Station: henan province people's Software Ver: 3.0.1.8

Report Date: 10/15/2018 11:41:24 AM Page: 1

Image Analysis Report

ZEISS

OS

Visante™ OCT
ANTERIOR SEGMENT IMAGING

Patient name: j

Date of birth: 9/12/1983
Patient ID: A15757

Exam date: 10/15/2018 11:33:37 AM

Rx: Sphere: 0.0
Cylinder: 0.0
Axis: 0.0
Fixation Angle: 0.0
Polarization: 33.0
Orientation: 0.0

Protocol: LVC Pachymetry And Topography
Scan: Enhanced High Res. Corneal

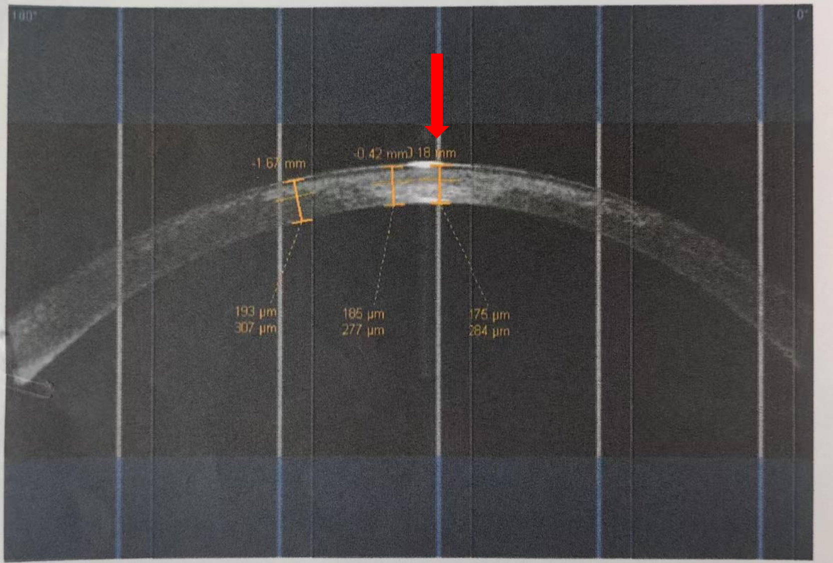

Planning

Institution:
Planner: operator

Date: 10/15/2018 11:41:57 AM Serial Number: 1000-2193
Station: henan province people's Software Ver: 3.0.1.8

Acquisition

Institution:
Operator: operator

Serial Number: 1000-2193
Station: henan province people's Software Ver: 3.0.1.8

Report Date: 10/15/2018 11:42:47 AM Page: 1

N6

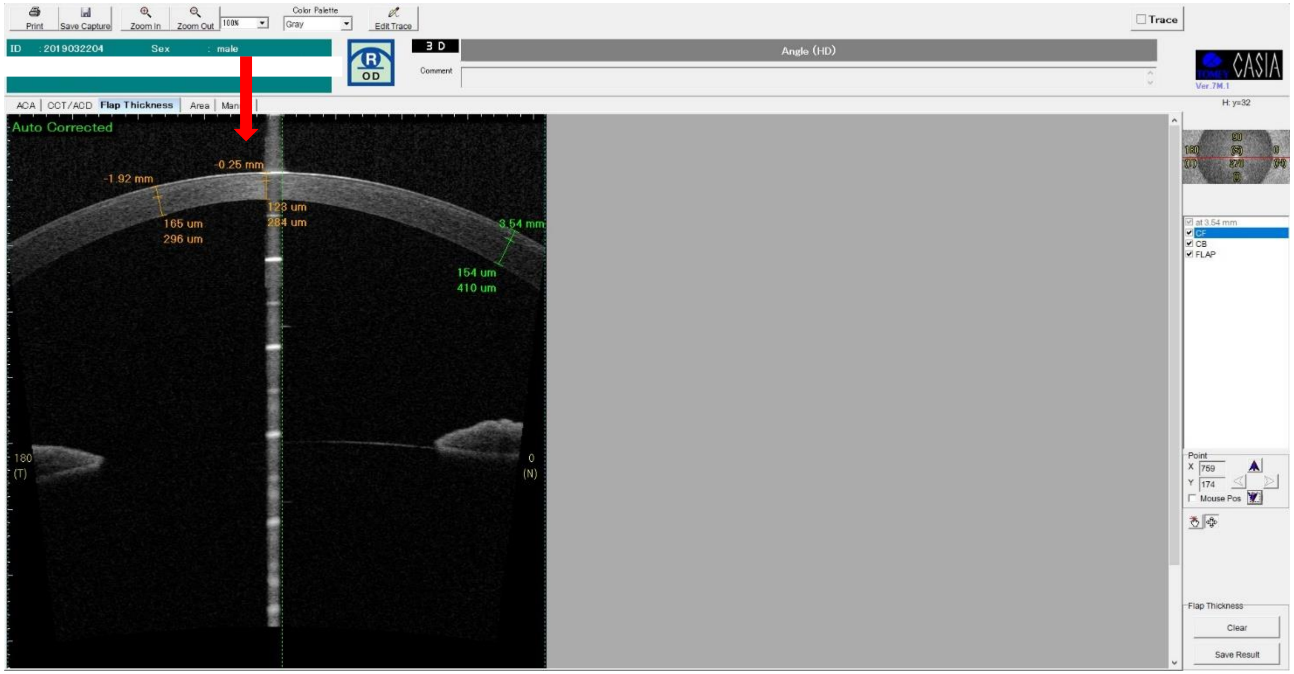
Print
Save Capture
Zoom In
Zoom Out
Color Palette
Gray
Edit Trace
Trace
ID : 2019032204 Sex : male
OD
3 D
Angle (HD)
Comment
CASIA
ACA | CCT/ACD | Flap Thickness | Area | Man
Auto Corrected
-0.25 mm
-1.92 mm
123 um
284 um
165 um
296 um
3.54 mm
154 um
410 um
180
(T)
0
(N)
CF
CB
FLAP
Point
Mouse Pos
Flap Thickness
Clear
Save Result

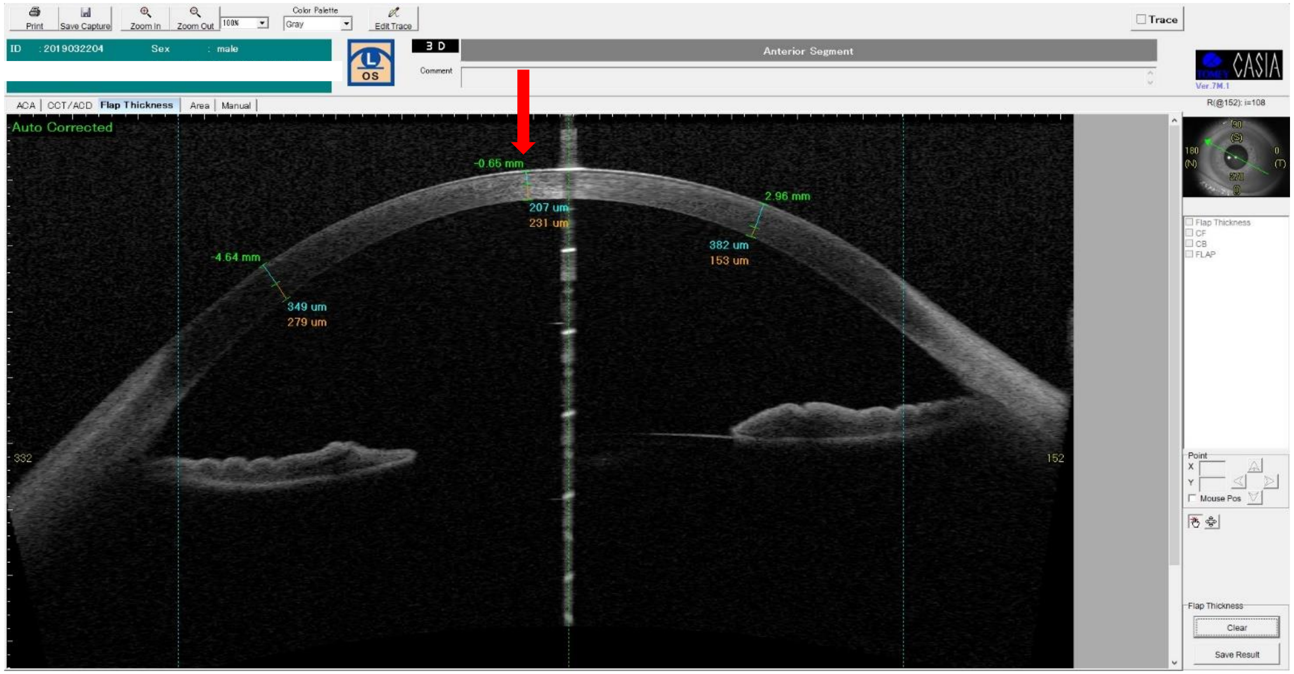
Print
Save Capture
Zoom In
Zoom Out
Color Palette
Gray
Edit Trace
Trace
ID : 2019032204 Sex : male
OS
3 D
Anterior Segment
Comment
CASIA
ACA | CCT/ACD | Flap Thickness | Area | Manual
Auto Corrected
-0.65 mm
207 um
231 um
2.96 mm
382 um
153 um
-4.64 mm
349 um
279 um
332
152
Flap Thickness
CF
CB
FLAP
Point
Mouse Pos
Clear
Save Result

N7

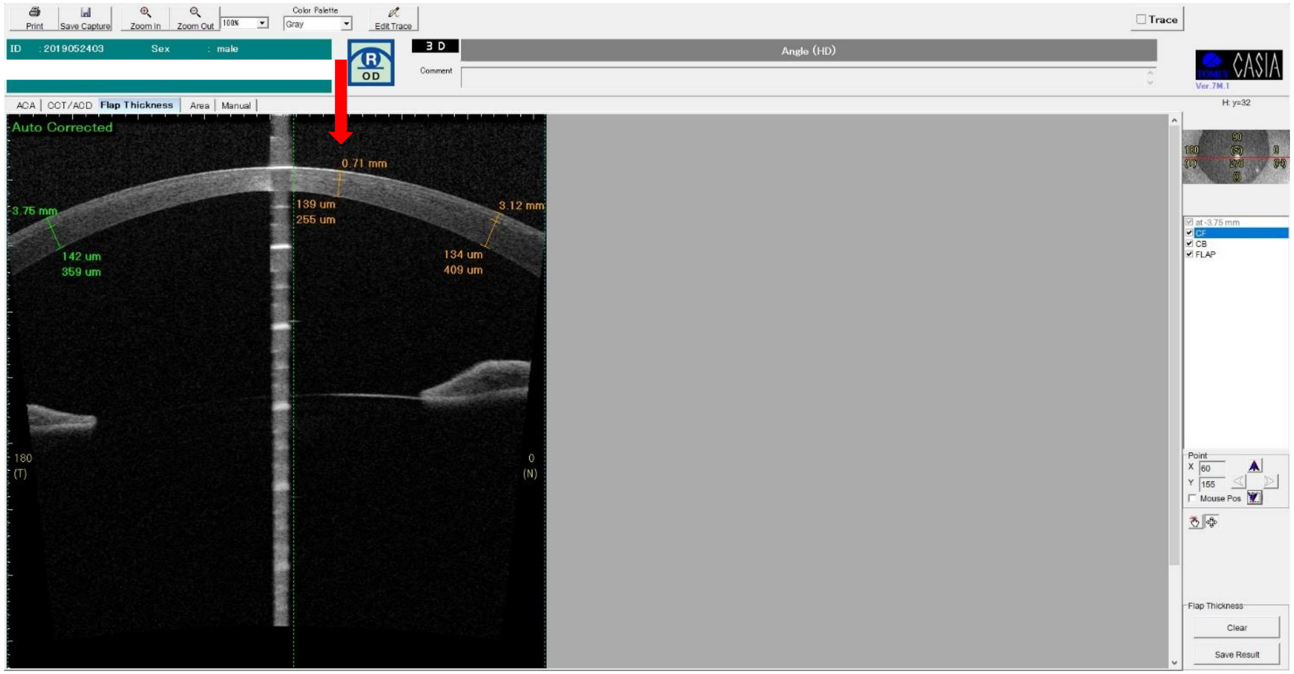

ID : 2019052403
Sex : male
OD
3 D
Angle (HD)
ACA
CCT/ACD
Flap Thickness
Area
Manual
Auto Corrected
0.71 mm
139 um
255 um
3.75 mm
142 um
359 um
3.12 mm
134 um
409 um
180
(T)
0
(N)
CASIA
Flap Thickness
Clear
Save Result

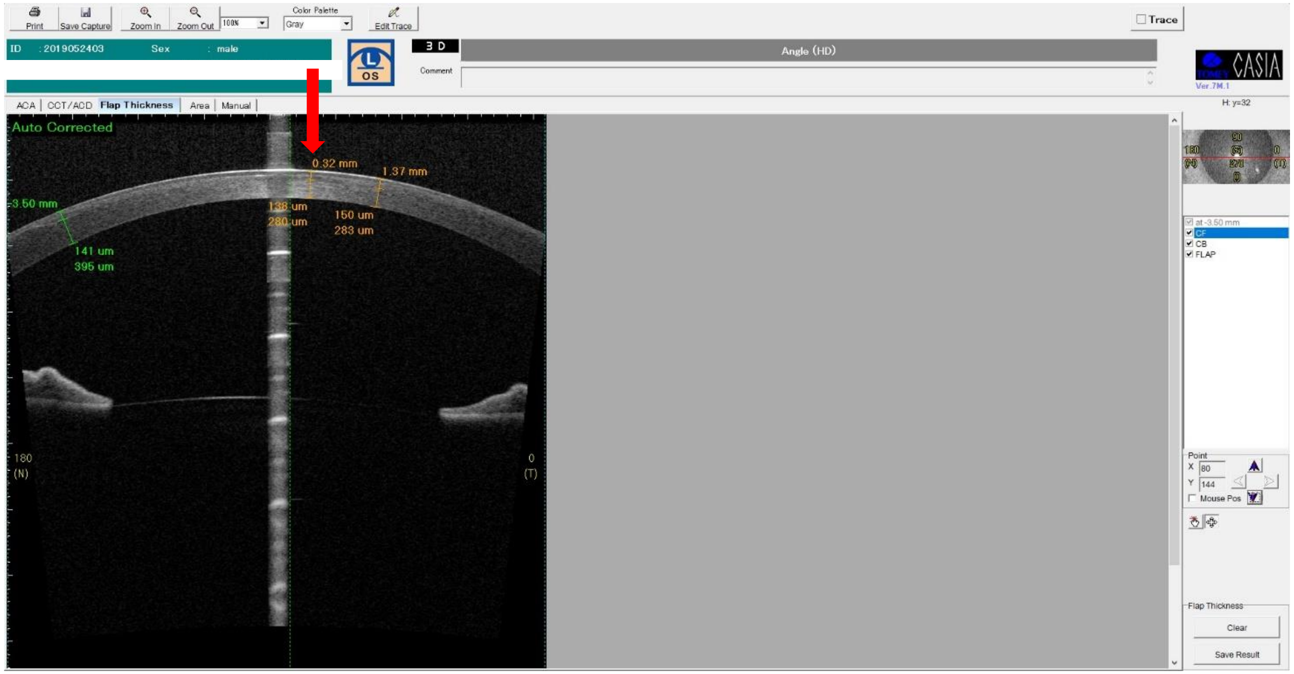

ID : 2019052403
Sex : male
OS
3 D
Angle (HD)
ACA
CCT/ACD
Flap Thickness
Area
Manual
Auto Corrected
0.32 mm
1.37 mm
3.50 mm
138 um
280 um
150 um
283 um
141 um
395 um
180
(N)
0
(T)
CASIA
Flap Thickness
Clear
Save Result

N8

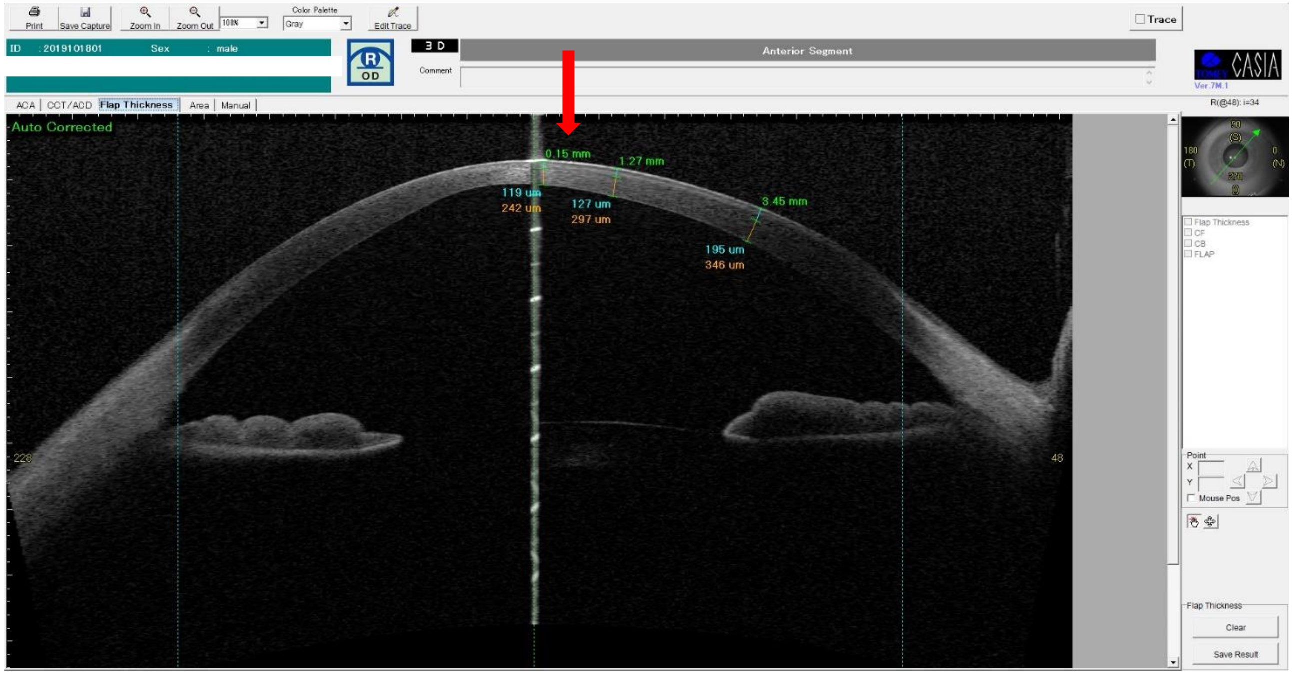
Print
Save Capture
Zoom In
Zoom Out
100%
Color Palette
Gray
Edit Trace
Trace
ID : 2019101801
Sex : male
R
OD
3 D
Anterior Segment
Comment
CASIA
ACA
CCT/ACD
Flap Thickness
Area
Manual
R(@48): i=34
Auto Corrected
0.15 mm
1.27 mm
119 um
242 um
127 um
297 um
3.45 mm
195 um
346 um
228
48
Flap Thickness
CF
CB
FLAP
Point
X
Y
Mouse Pos
Flap Thickness
Clear
Save Result

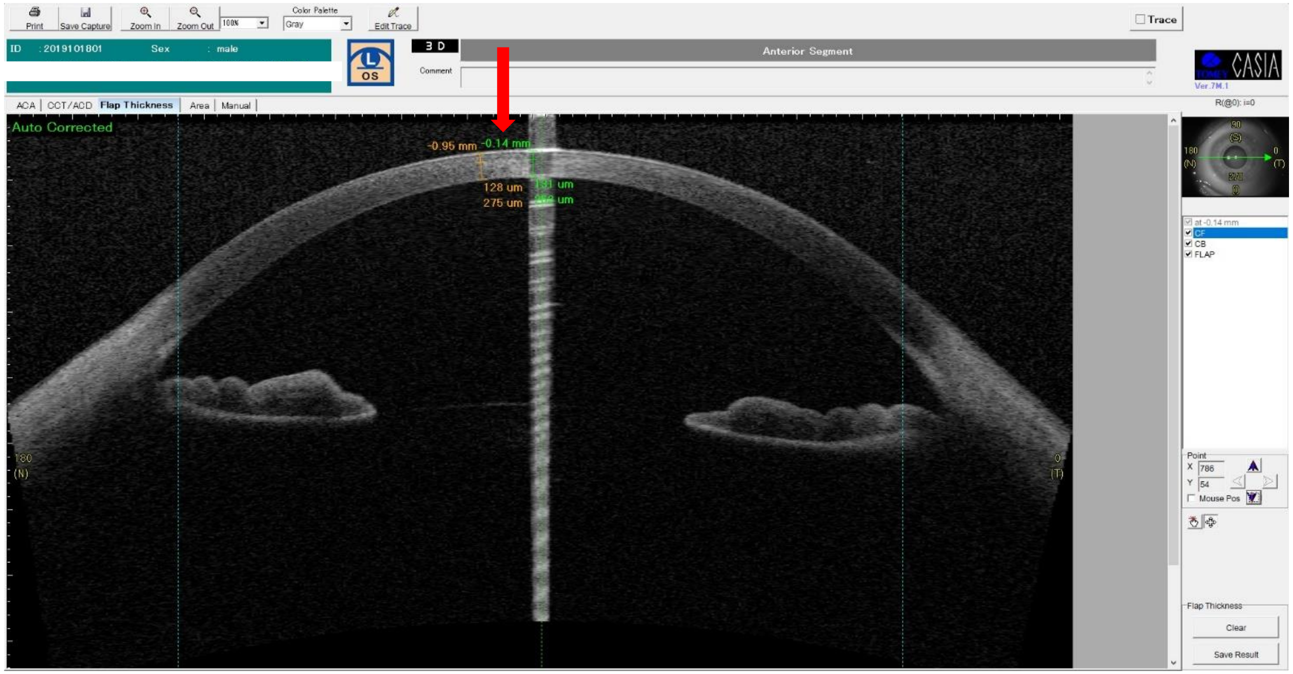
Print
Save Capture
Zoom In
Zoom Out
100%
Color Palette
Gray
Edit Trace
Trace
ID : 2019101801
Sex : male
L
OS
3 D
Anterior Segment
Comment
CASIA
ACA
CCT/ACD
Flap Thickness
Area
Manual
R(@0): i=0
Auto Corrected
-0.95 mm
-0.14 mm
128 um
275 um
180
(N)
0
(T)
at -0.14 mm
CF
CB
FLAP
Point
X 786
Y 54
Mouse Pos
Flap Thickness
Clear
Save Result

N9

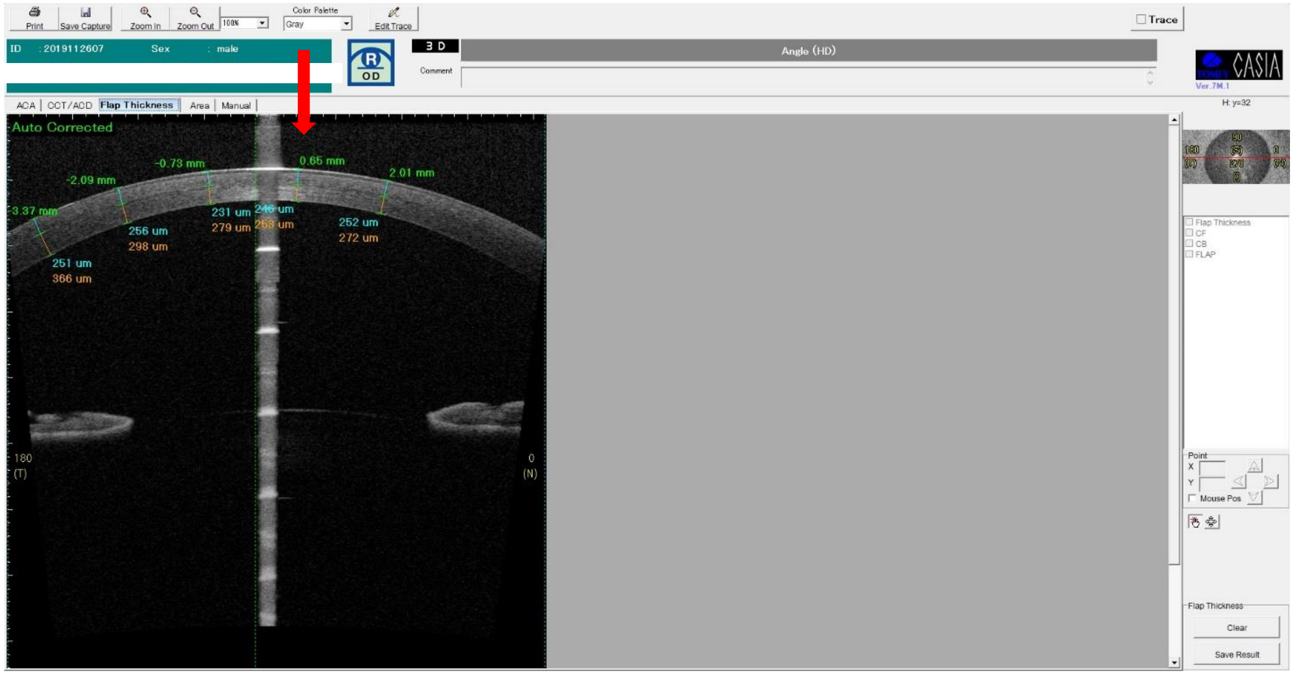

Print
Save Capture
Zoom In
Zoom Out
Color Palette
Gray
Edit Trace
Trace
ID : 2019112607 Sex : male
OD
3 D
Comment
Angle (HD)
CASIA
ACA | OCT/ACD | Flap Thickness | Area | Manual
Auto Corrected
-0.73 mm
0.65 mm
-2.09 mm
2.01 mm
-3.37 mm
231 um
279 um
246 um
263 um
256 um
298 um
252 um
272 um
251 um
366 um
180
(T)
0
(N)
Flap Thickness
CF
CB
FLAP
Point
Mouse Pos
Clear
Save Result

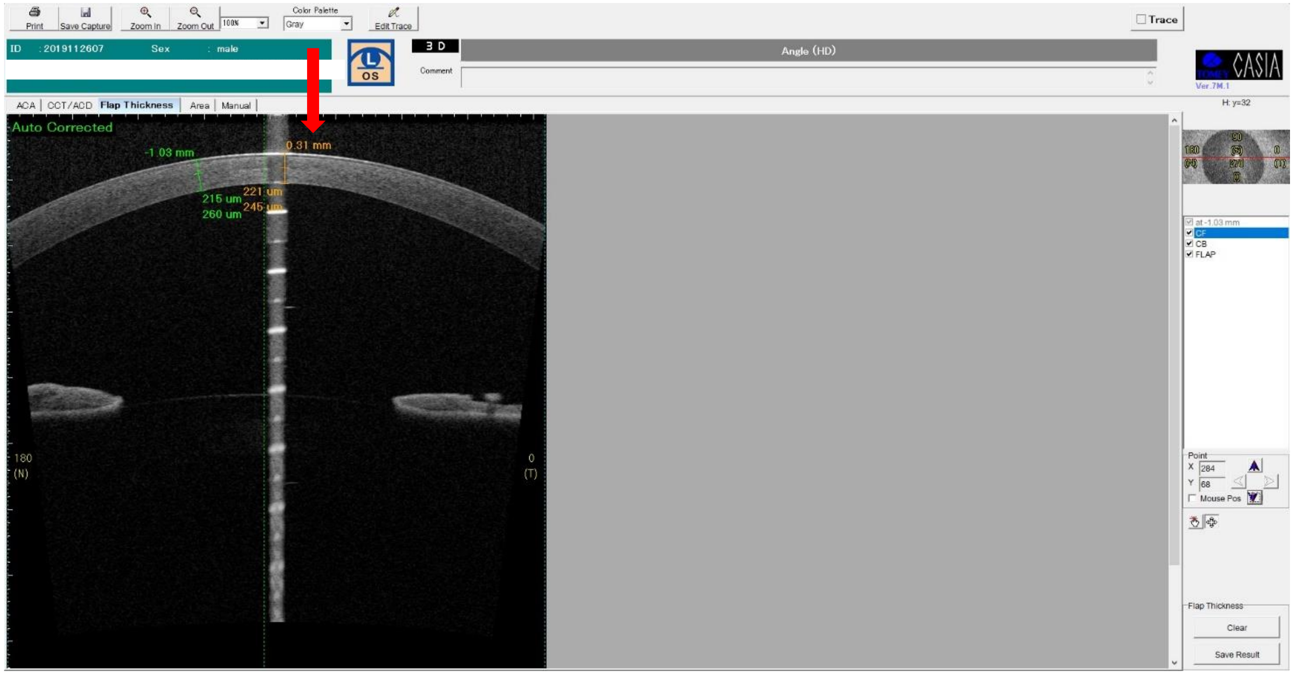

Print
Save Capture
Zoom In
Zoom Out
Color Palette
Gray
Edit Trace
Trace
ID : 2019112607 Sex : male
OS
3 D
Comment
Angle (HD)
CASIA
ACA | OCT/ACD | Flap Thickness | Area | Manual
Auto Corrected
-1.03 mm
0.31 mm
215 um
260 um
221 um
245 um
180
(N)
0
(T)
CF
CB
FLAP
Point
284
68
Mouse Pos
Clear
Save Result

N10

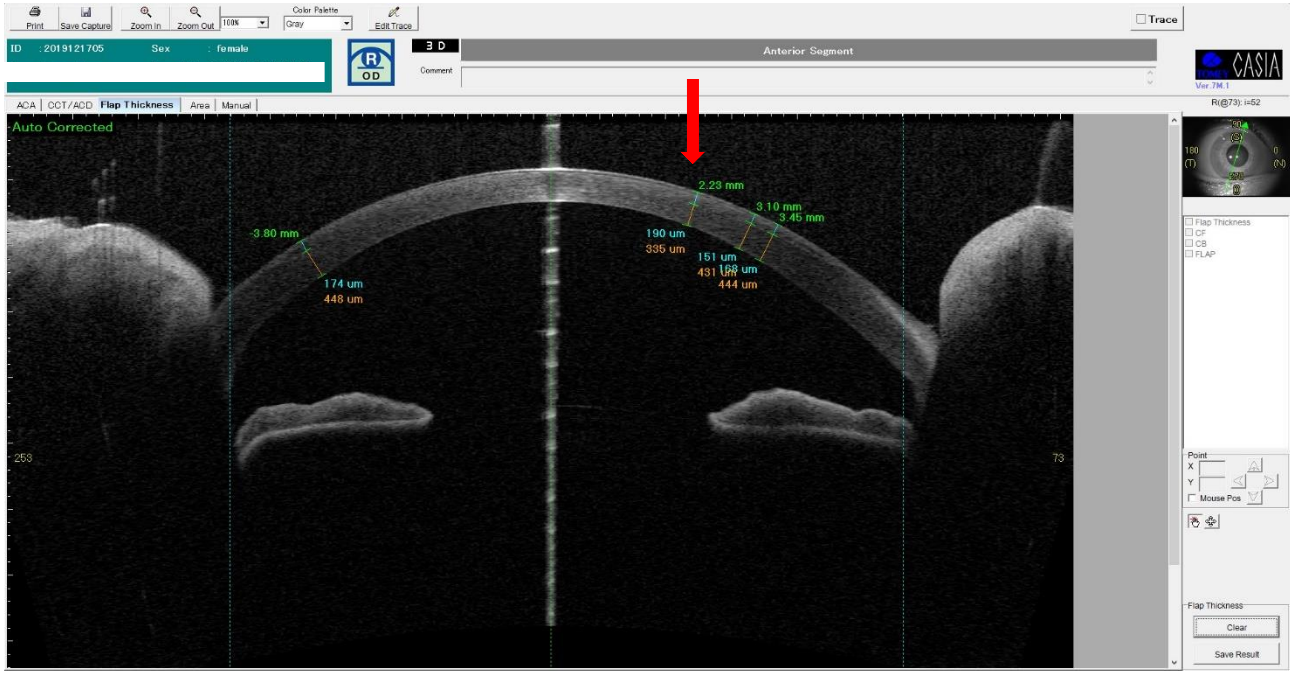

Print
Save Capture
Zoom In
Zoom Out
100%
Color Palette
Gray
Edit Trace
Trace
ID : 2019121705
Sex : female
3 D
Anterior Segment
Comment
CASIA
ACA
CCT/ACD
Flap Thickness
Area
Manual
Auto Corrected
2.23 mm
3.10 mm
3.45 mm
-3.80 mm
190 um
335 um
151 um
444 um
174 um
448 um
253
73
Point
Mouse Pos
Flap Thickness
Clear
Save Result

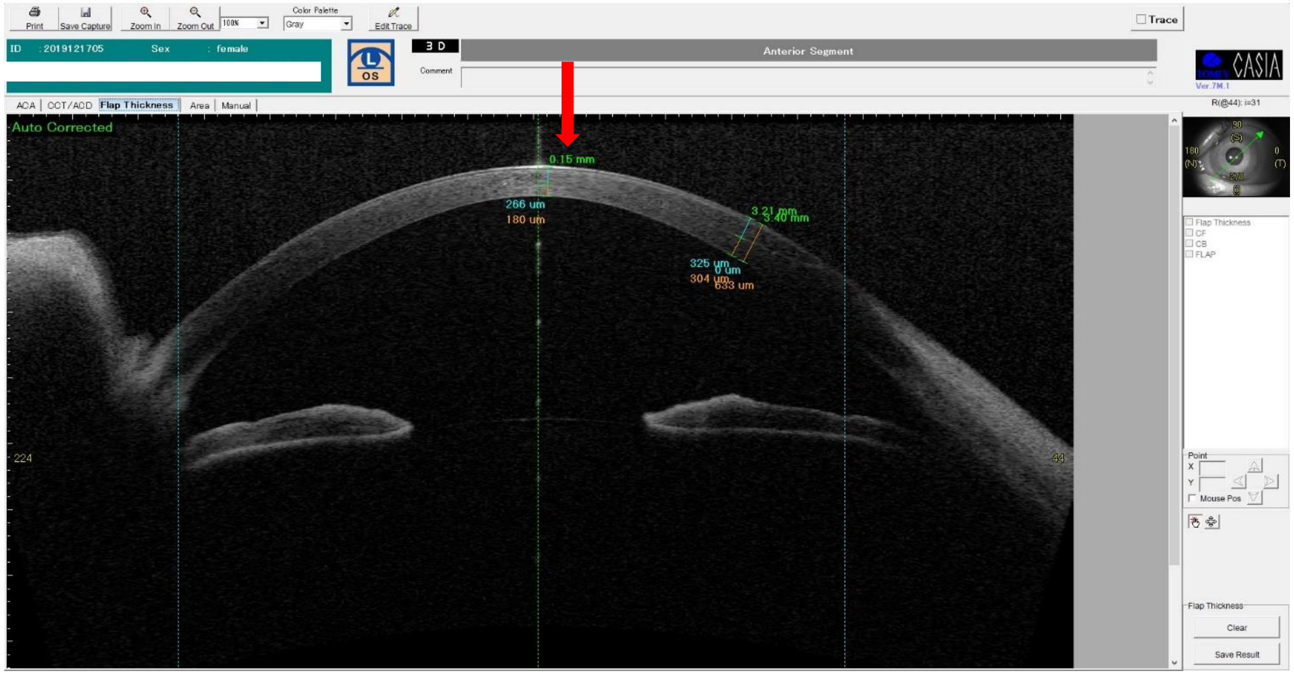

Print
Save Capture
Zoom In
Zoom Out
100%
Color Palette
Gray
Edit Trace
Trace
ID : 2019121705
Sex : female
3 D
Anterior Segment
Comment
CASIA
ACA
CCT/ACD
Flap Thickness
Area
Manual
Auto Corrected
0.15 mm
266 um
180 um
3.21 mm
3.40 mm
325 um
304 um
633 um
224
44
Point
Mouse Pos
Flap Thickness
Clear
Save Result

N11

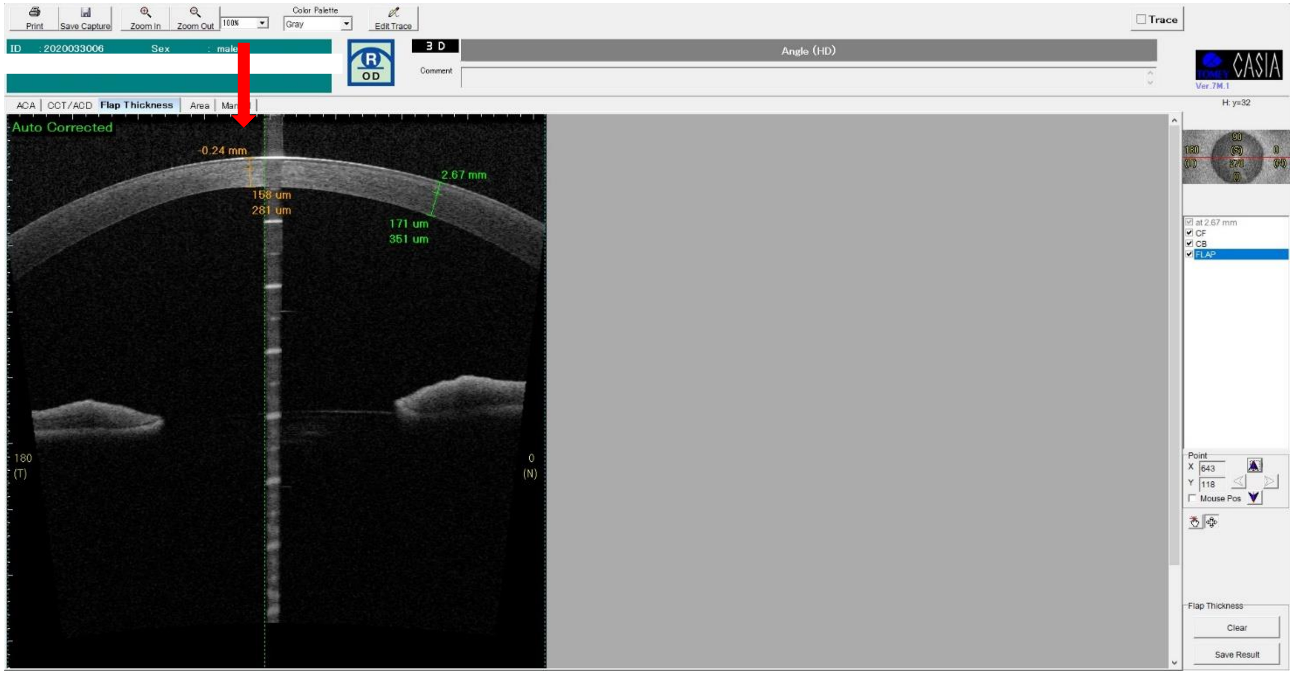
Print
Save Capture
Zoom In
Zoom Out
Color Palette
Gray
Edit Trace
Trace
ID : 2020033006
Sex : male
OD
3 D
Angle (HD)
Comment
CASIA
ACA
CCT/ACD
Flap Thickness
Area
Auto Corrected
-0.24 mm
158 um
281 um
2.67 mm
171 um
351 um
180
(T)
0
(N)
at 2.67 mm
CF
CB
FLAP
Point
X 643
Y 118
Mouse Pos
Flap Thickness
Clear
Save Result

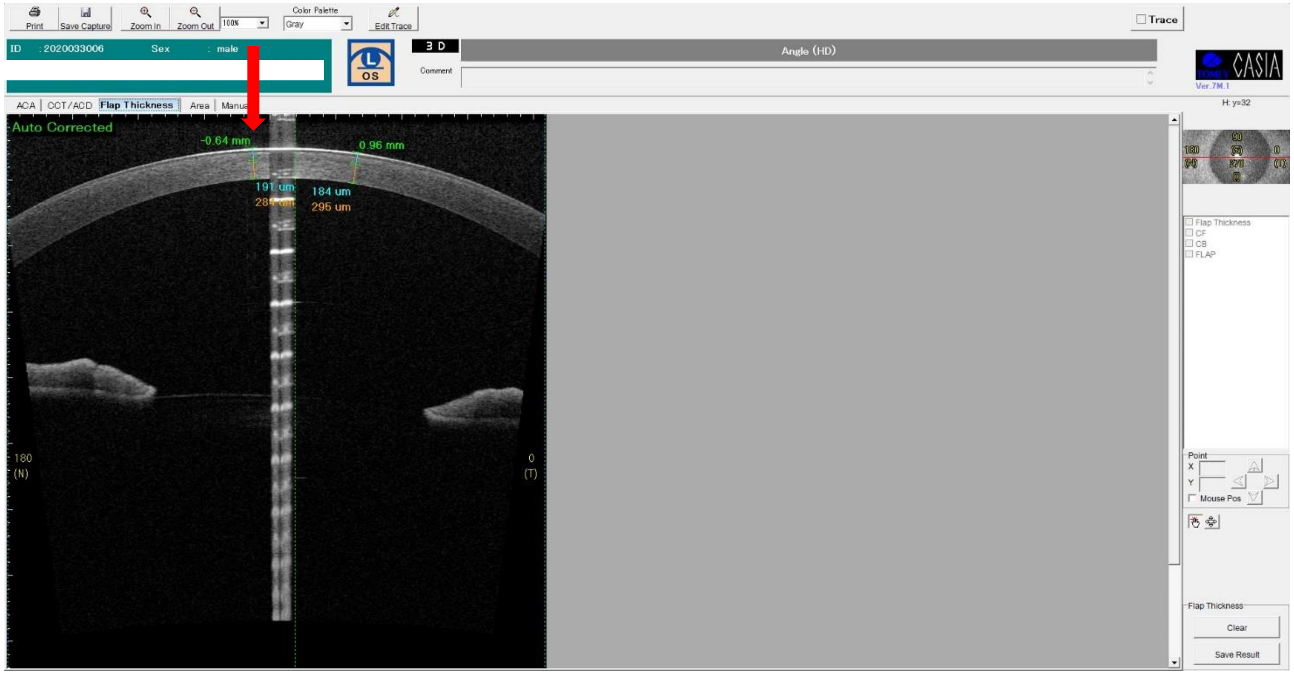
Print
Save Capture
Zoom In
Zoom Out
Color Palette
Gray
Edit Trace
Trace
ID : 2020033006
Sex : male
OS
3 D
Angle (HD)
Comment
CASIA
ACA
CCT/ACD
Flap Thickness
Area
Auto Corrected
-0.64 mm
191 um
0.96 mm
184 um
295 um
180
(N)
0
(T)
Flap Thickness
CF
CB
FLAP
Point
Mouse Pos
Flap Thickness
Clear
Save Result

N12

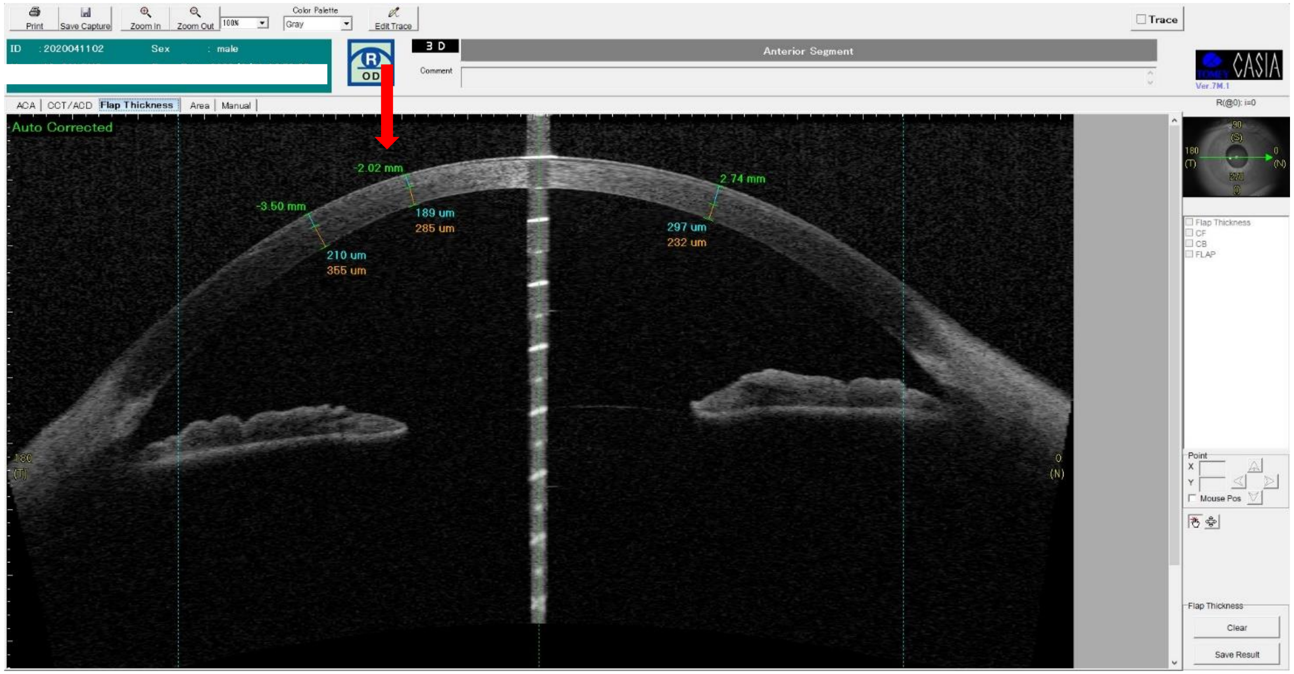
Print
Save Capture
Zoom In
Zoom Out
Color Palette
Gray
Edit Trace
Trace
ID : 2020041102
Sex : male
R
OD
3 D
Comment
Anterior Segment
CASIA
ACA
CCT/ACD
Flap Thickness
Area
Manual
Auto Corrected
-2.02 mm
-3.50 mm
189 um
285 um
210 um
355 um
2.74 mm
297 um
232 um
180 (T)
0 (N)
Flap Thickness
CF
CB
FLAP
Point
Mouse Pos
Clear
Save Result

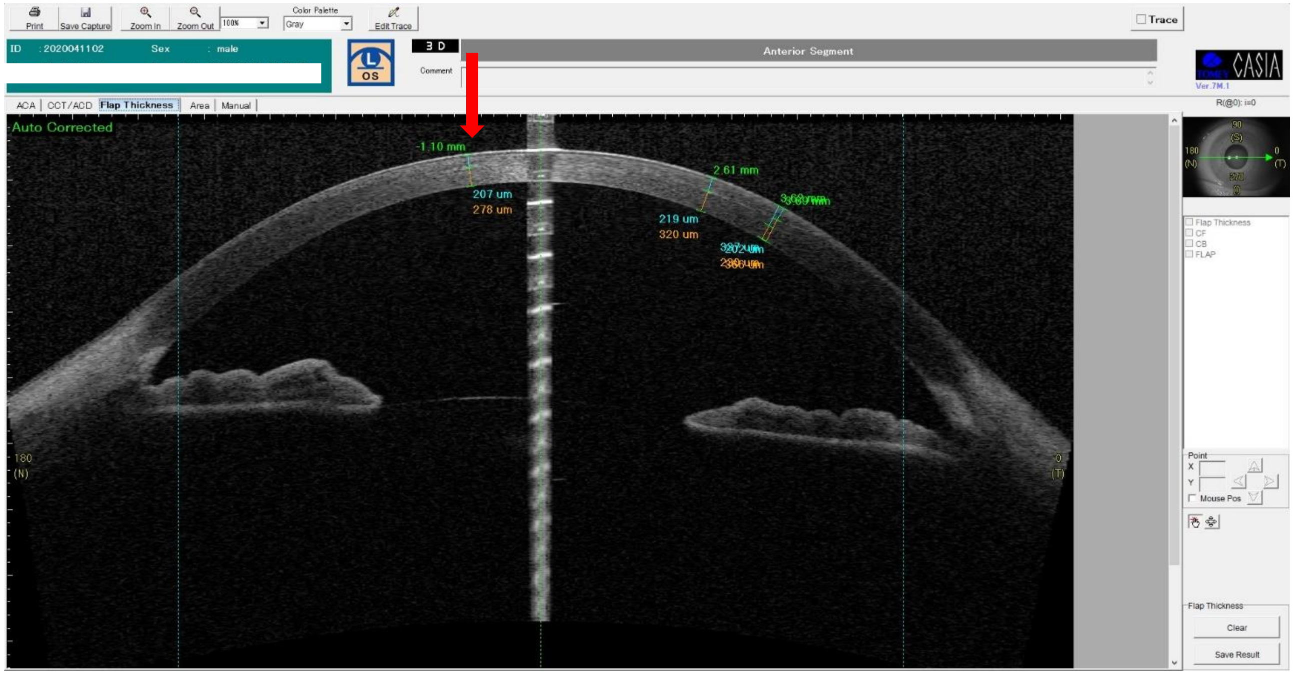
Print
Save Capture
Zoom In
Zoom Out
Color Palette
Gray
Edit Trace
Trace
ID : 2020041102
Sex : male
L
OS
3 D
Comment
Anterior Segment
CASIA
ACA
CCT/ACD
Flap Thickness
Area
Manual
Auto Corrected
-1.10 mm
207 um
278 um
2.61 mm
219 um
320 um
180 (N)
0 (T)
Flap Thickness
CF
CB
FLAP
Point
Mouse Pos
Clear
Save Result

N13

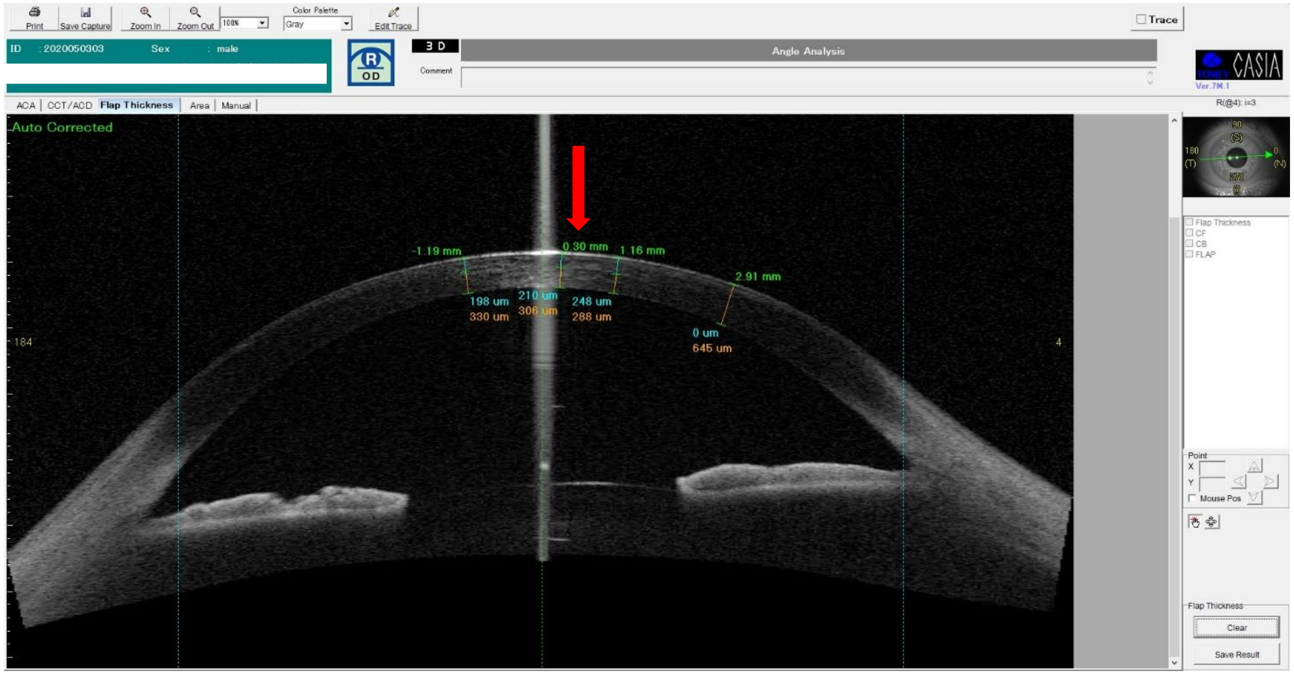
Print
Save Capture
Zoom In
Zoom Out
Color Palette
Gray
Edit Trace
Trace
ID : 2020050303 Sex : male
3 D
Angle Analysis
Comment
CASIA
ACA | OCT/ACD Flap Thickness Area Manual
Auto Corrected
-1.19 mm
0.30 mm
1.16 mm
2.91 mm
198 um
330 um
210 um
306 um
248 um
288 um
0 um
645 um
184
4
Flap Thickness
CF
CB
FLAP
Point
Mouse Pos
Clear
Save Result

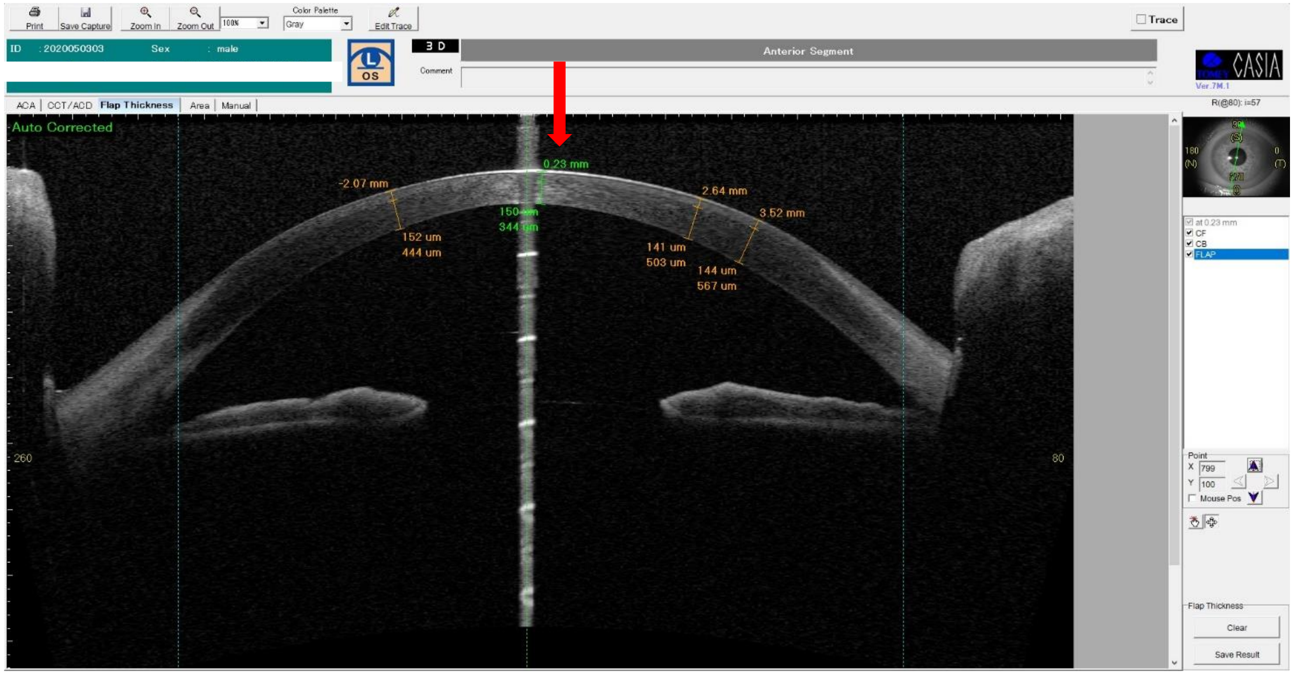
Print
Save Capture
Zoom In
Zoom Out
Color Palette
Gray
Edit Trace
Trace
ID : 2020050303 Sex : male
3 D
Anterior Segment
Comment
CASIA
ACA | OCT/ACD Flap Thickness Area Manual
Auto Corrected
-2.07 mm
0.23 mm
2.64 mm
3.52 mm
152 um
444 um
150 um
344 um
141 um
503 um
144 um
567 um
260
80
at 0.23 mm
CF
CB
FLAP
Point
X 799
Y 100
Mouse Pos
Clear
Save Result

N14

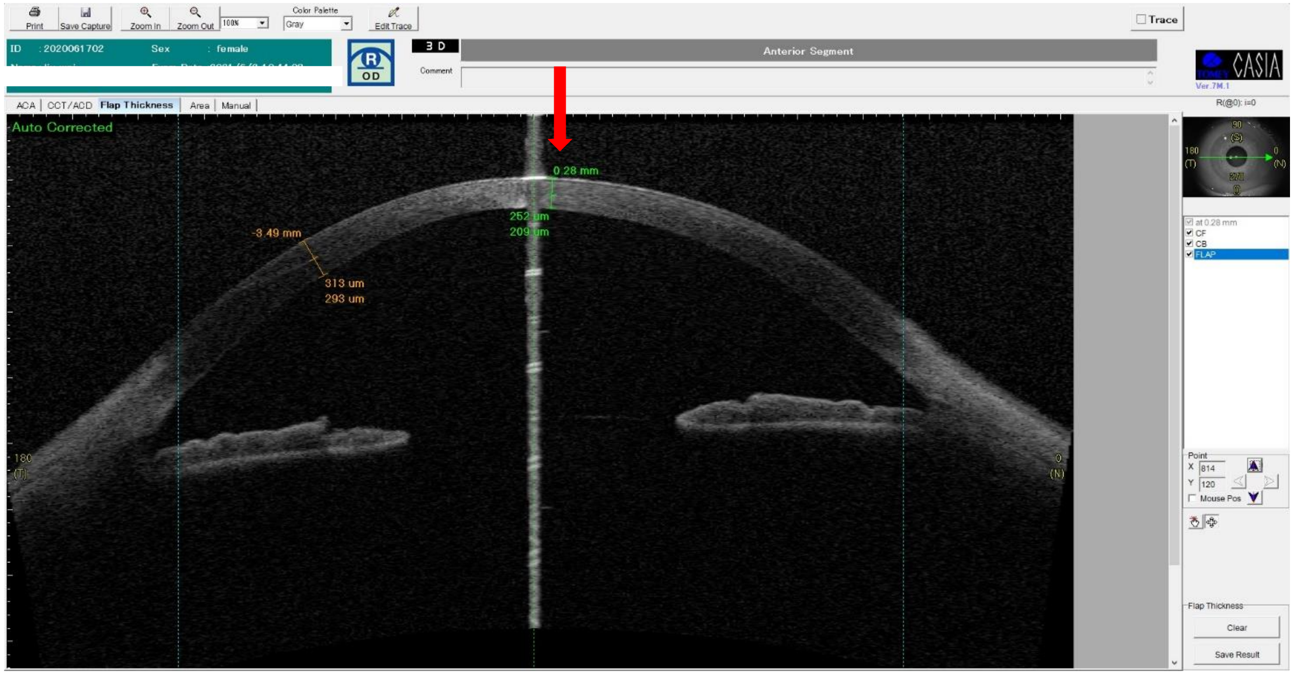
Print
Save Capture
Zoom In
Zoom Out
Color Palette
Gray
Edit Trace
Trace
ID : 2020061702
Sex : female
R
OD
3 D
Comment
Anterior Segment
CASIA
ACA
CCT/ACD
Flap Thickness
Area
Manual
Auto Corrected
0.28 mm
252 um
209 um
-3.49 mm
313 um
293 um
180
(T)
0
(N)
CF
CB
FLAP
Point
Mouse Pos
Flap Thickness
Clear
Save Result

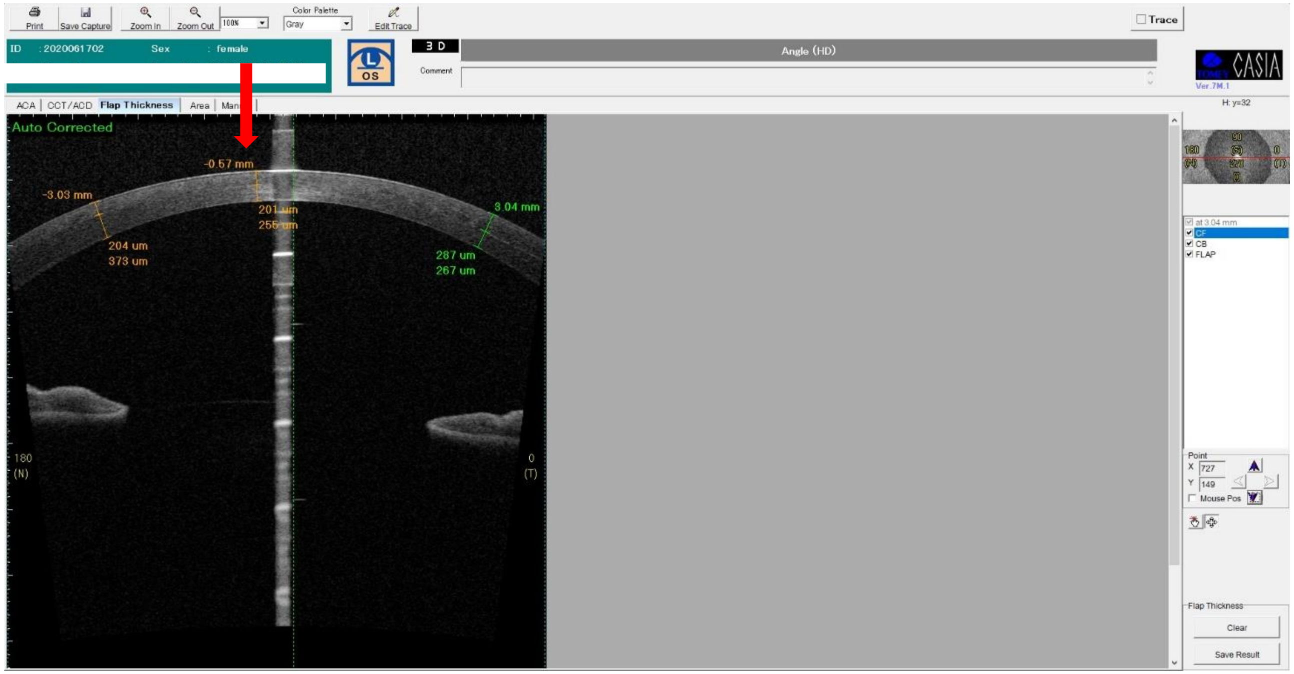
Print
Save Capture
Zoom In
Zoom Out
Color Palette
Gray
Edit Trace
Trace
ID : 2020061702
Sex : female
L
OS
3 D
Comment
Angle (HD)
CASIA
ACA
CCT/ACD
Flap Thickness
Area
Auto Corrected
-0.57 mm
201 um
255 um
-3.03 mm
204 um
373 um
3.04 mm
287 um
267 um
180
(N)
0
(T)
CF
CB
FLAP
Point
Mouse Pos
Flap Thickness
Clear
Save Result

N15

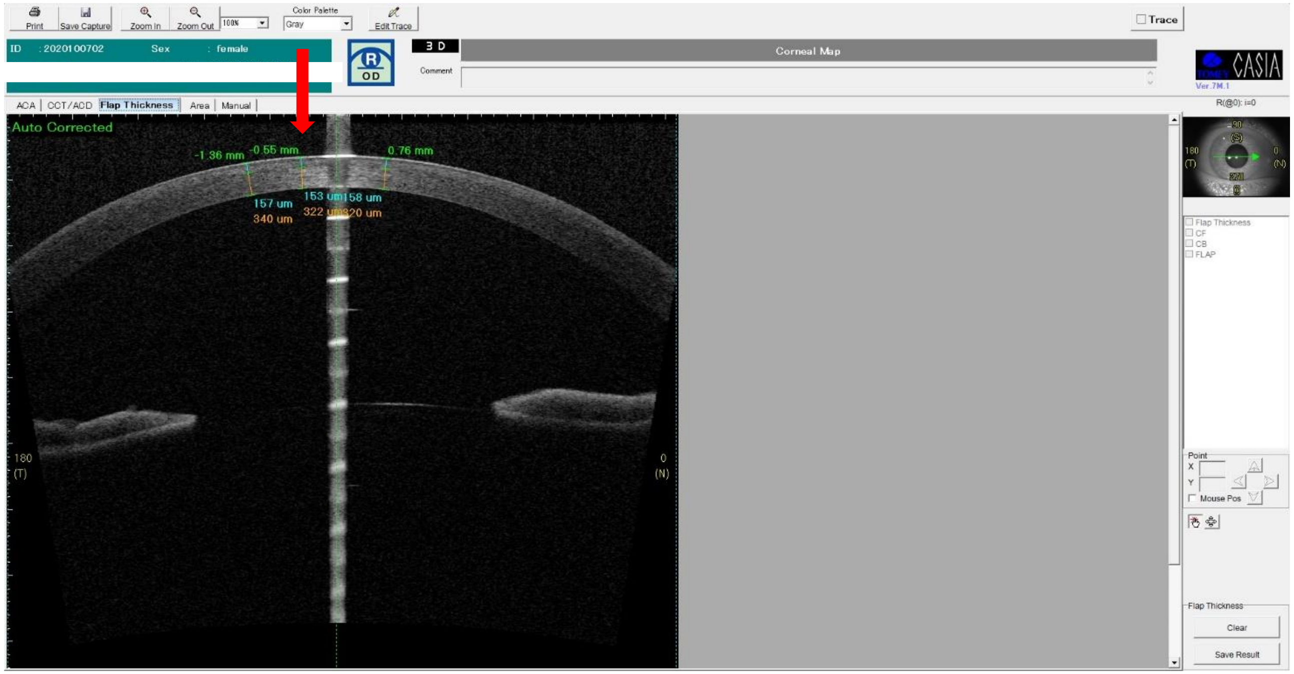
Print
Save Capture
Zoom In
Zoom Out
100%
Color Palette
Gray
Edit Trace
Trace
ID : 2020100702
Sex : female
R
OD
3 D
Corneal Map
Comment
CASIA
Ver.7M.1
ACA
OCT/ACD
Flap Thickness
Area
Manual
Auto Corrected
-1.36 mm
-0.55 mm
0.76 mm
157 um
340 um
153 um
322 um
158 um
320 um
180
(T)
0
(N)
R(@0): i=0
Flap Thickness
CF
CB
FLAP
Point
X
Y
Mouse Pos
Clear
Save Result

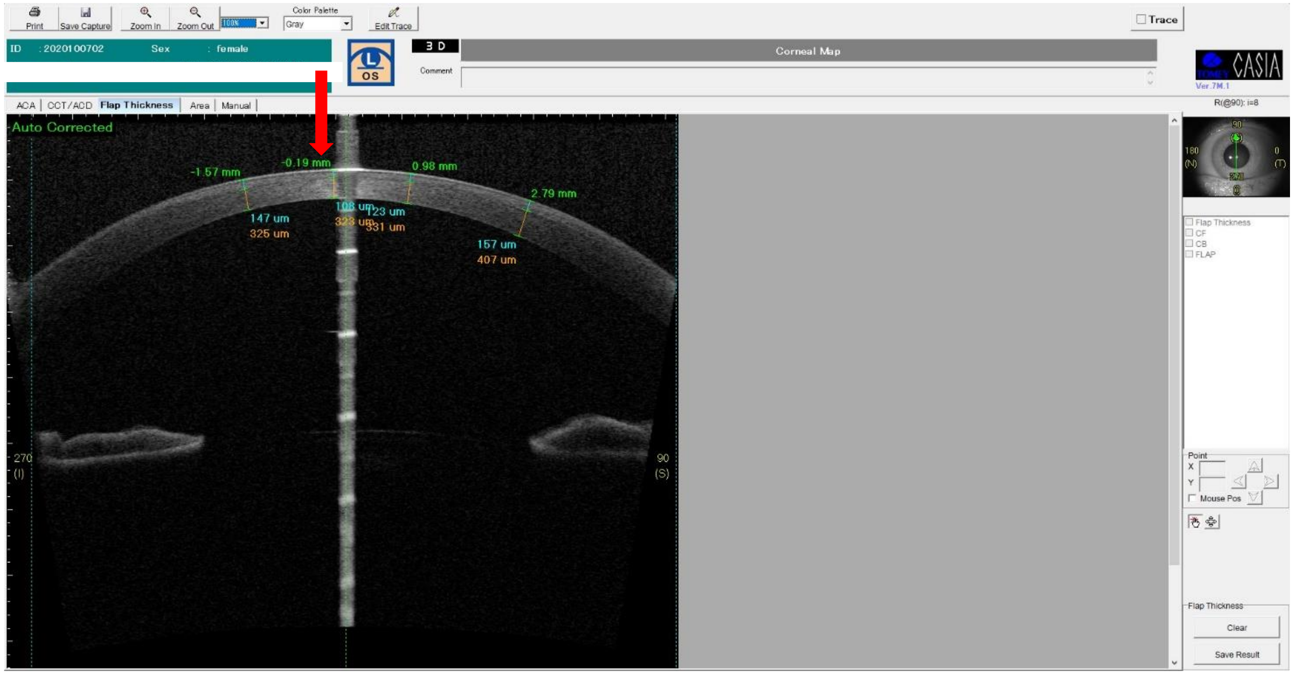
Print
Save Capture
Zoom In
Zoom Out
100%
Color Palette
Gray
Edit Trace
Trace
ID : 2020100702
Sex : female
L
OS
3 D
Corneal Map
Comment
CASIA
Ver.7M.1
ACA
OCT/ACD
Flap Thickness
Area
Manual
Auto Corrected
-1.57 mm
-0.19 mm
0.98 mm
2.79 mm
147 um
325 um
108 um
323 um
123 um
331 um
157 um
407 um
270
(I)
90
(S)
R(@90): i=8
Flap Thickness
CF
CB
FLAP
Point
X
Y
Mouse Pos
Clear
Save Result

N16

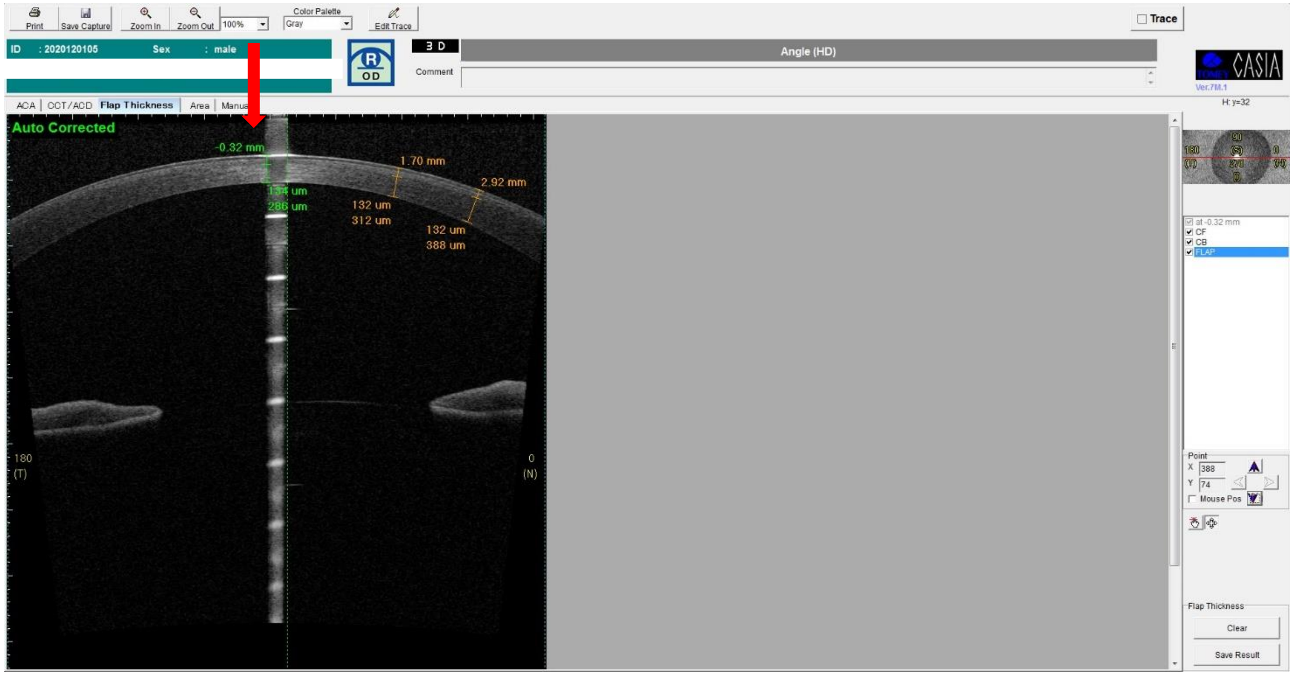

Print
Save Capture
Zoom In
Zoom Out
100%
Color Palette
Gray
Edit Trace
Trace
ID : 2020120105
Sex : male
R OD
3 D
Angle (HD)
Comment
CASIA
H: y=32
ACA
OCT/ACD
Flap Thickness
Area
Auto Corrected
-0.32 mm
1.70 mm
2.92 mm
134 um
286 um
132 um
312 um
132 um
388 um
180 (T)
0 (N)
CF
CB
FLAP
Point
X 388
Y 74
Mouse Pos
Flap Thickness
Clear
Save Result

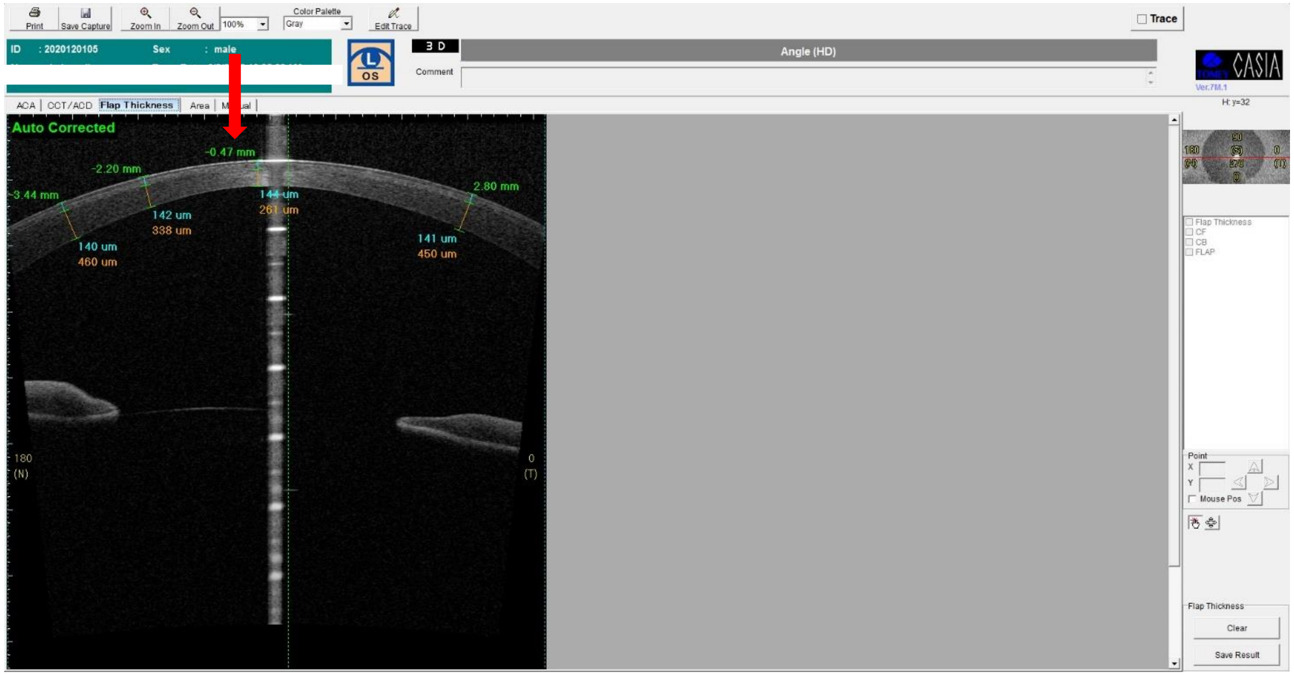

Print
Save Capture
Zoom In
Zoom Out
100%
Color Palette
Gray
Edit Trace
Trace
ID : 2020120105
Sex : male
L OS
3 D
Angle (HD)
Comment
CASIA
H: y=32
ACA
OCT/ACD
Flap Thickness
Area
Auto Corrected
-0.47 mm
-2.20 mm
-3.44 mm
2.80 mm
144 um
261 um
142 um
338 um
140 um
460 um
141 um
450 um
180 (N)
0 (T)
Flap Thickness
CF
CB
FLAP
Point
Mouse Pos
Flap Thickness
Clear
Save Result

N17

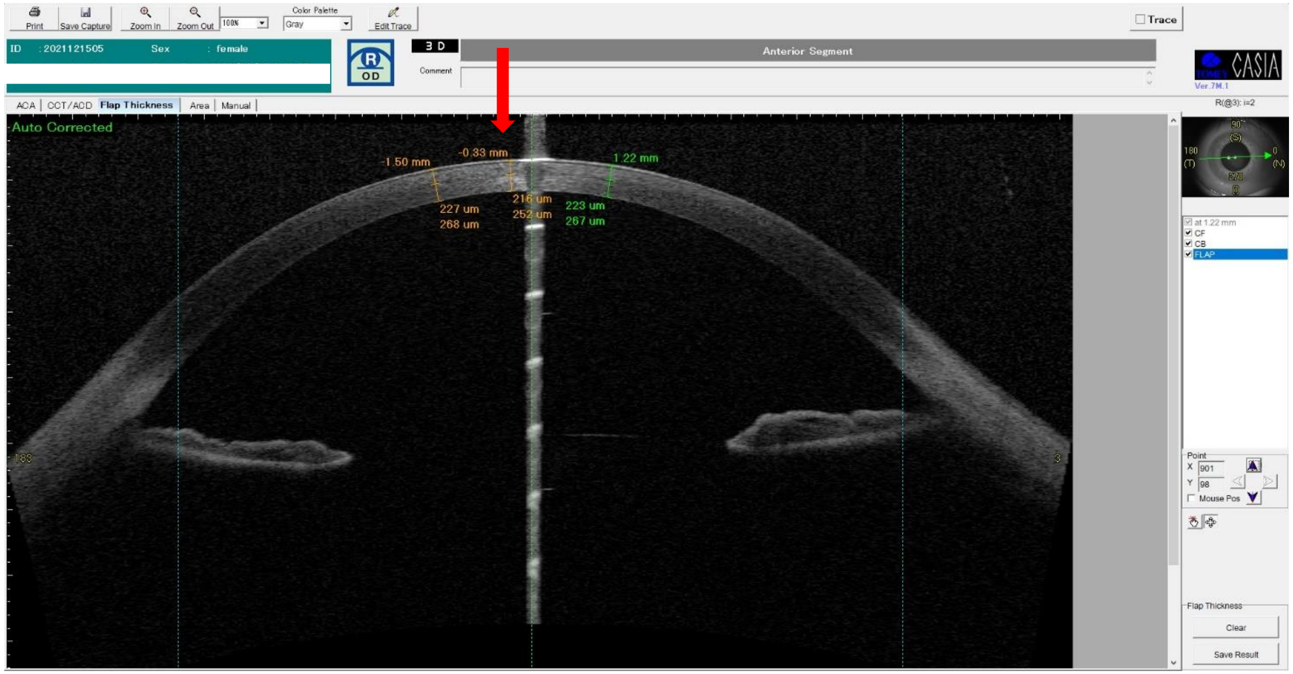

Print
Save Capture
Zoom In
Zoom Out
Color Palette
Gray
Edit Trace
Trace
ID : 2021121505
Sex : female
R OD
3 D
Anterior Segment
Comment
CASIA
ACA | OCT/ACD | Flap Thickness | Area | Manual
Auto Corrected
-1.50 mm
-0.33 mm
1.22 mm
227 um
268 um
216 um
252 um
223 um
267 um
at 1.22 mm
CF
CB
FLAP
Point
Mouse Pos
Flap Thickness
Clear
Save Result

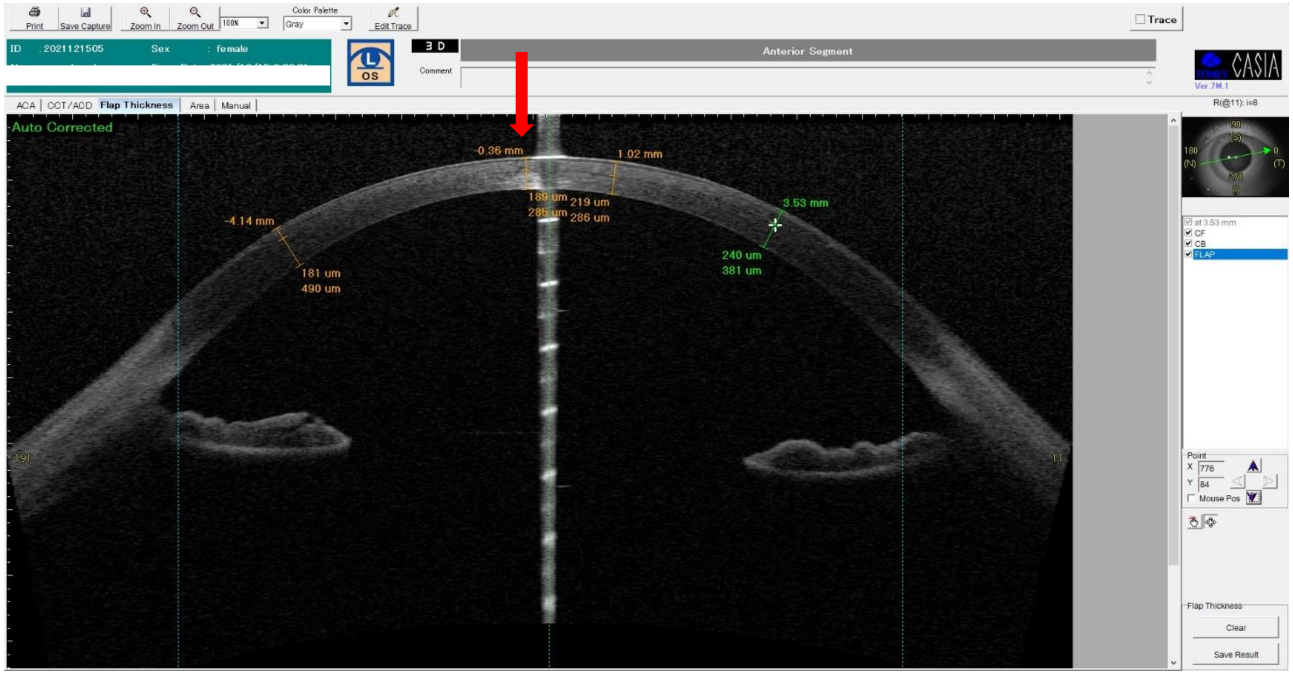

Print
Save Capture
Zoom In
Zoom Out
Color Palette
Gray
Edit Trace
Trace
ID : 2021121505
Sex : female
L OS
3 D
Anterior Segment
Comment
CASIA
ACA | OCT/ACD | Flap Thickness | Area | Manual
Auto Corrected
-0.36 mm
1.02 mm
-4.14 mm
3.53 mm
189 um
286 um
219 um
286 um
181 um
490 um
240 um
381 um
at 3.53 mm
CF
CB
FLAP
Point
Mouse Pos
Flap Thickness
Clear
Save Result

N18

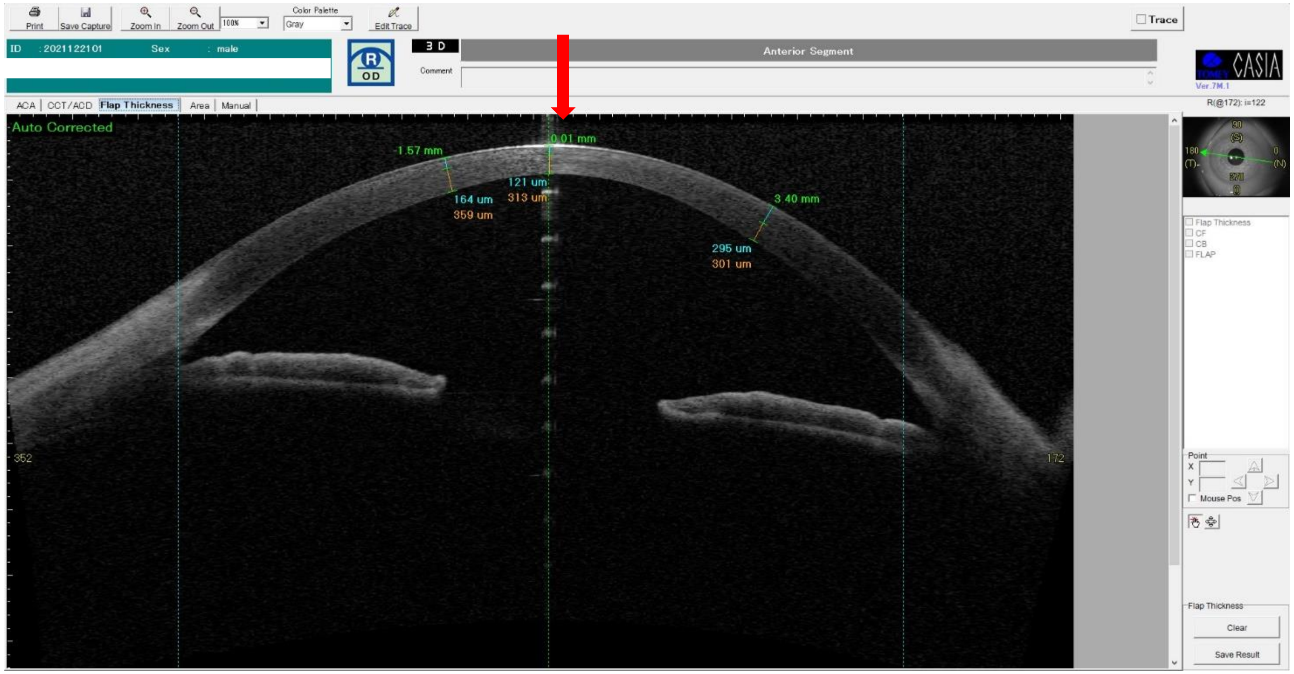
Print
Save Capture
Zoom In
Zoom Out
Color Palette
Gray
Edit Trace
Trace
ID : 2021122101 Sex : male
3 D
Anterior Segment
Comment
CASIA
OD
ACA | CCT/ACD | Flap Thickness | Area | Manual
Auto Corrected
-1.57 mm
0.01 mm
121 um
313 um
164 um
359 um
3.40 mm
295 um
301 um
352
172
Flap Thickness
Clear
Save Result

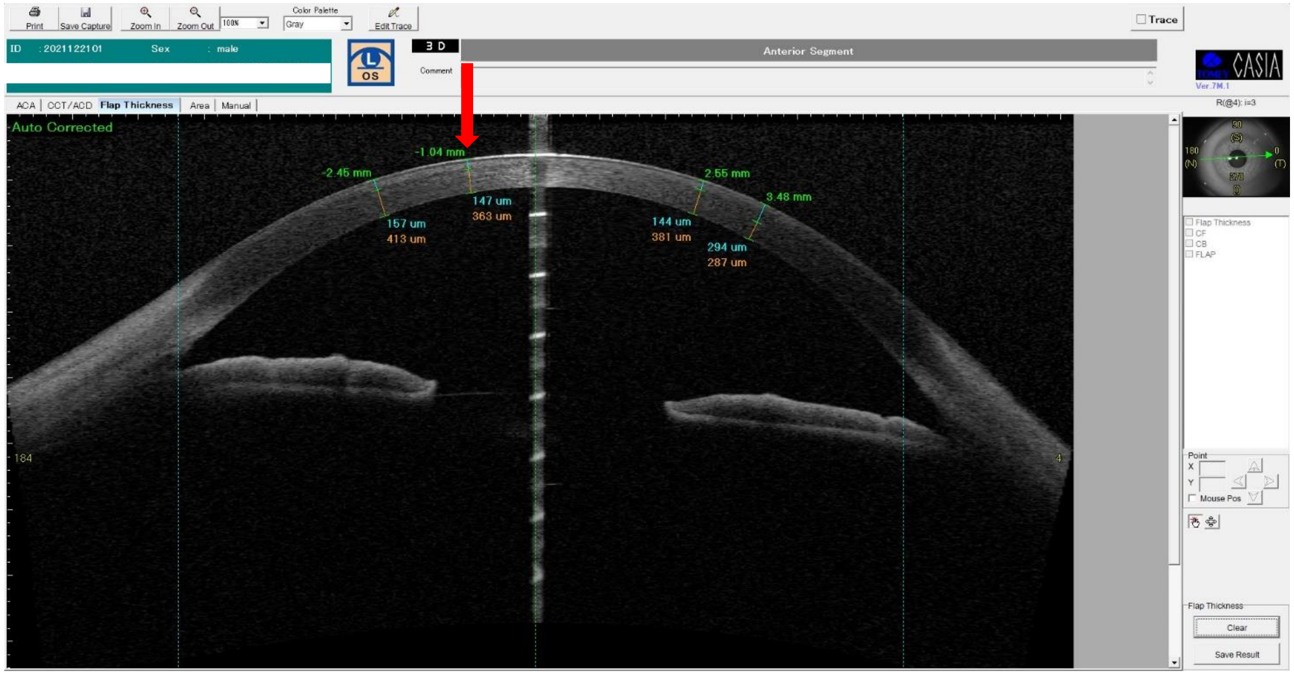
Print
Save Capture
Zoom In
Zoom Out
Color Palette
Gray
Edit Trace
Trace
ID : 2021122101 Sex : male
3 D
Anterior Segment
Comment
CASIA
OS
ACA | CCT/ACD | Flap Thickness | Area | Manual
Auto Corrected
-1.04 mm
-2.45 mm
147 um
363 um
157 um
413 um
2.55 mm
3.48 mm
144 um
381 um
294 um
287 um
184
Flap Thickness
Clear
Save Result

N19

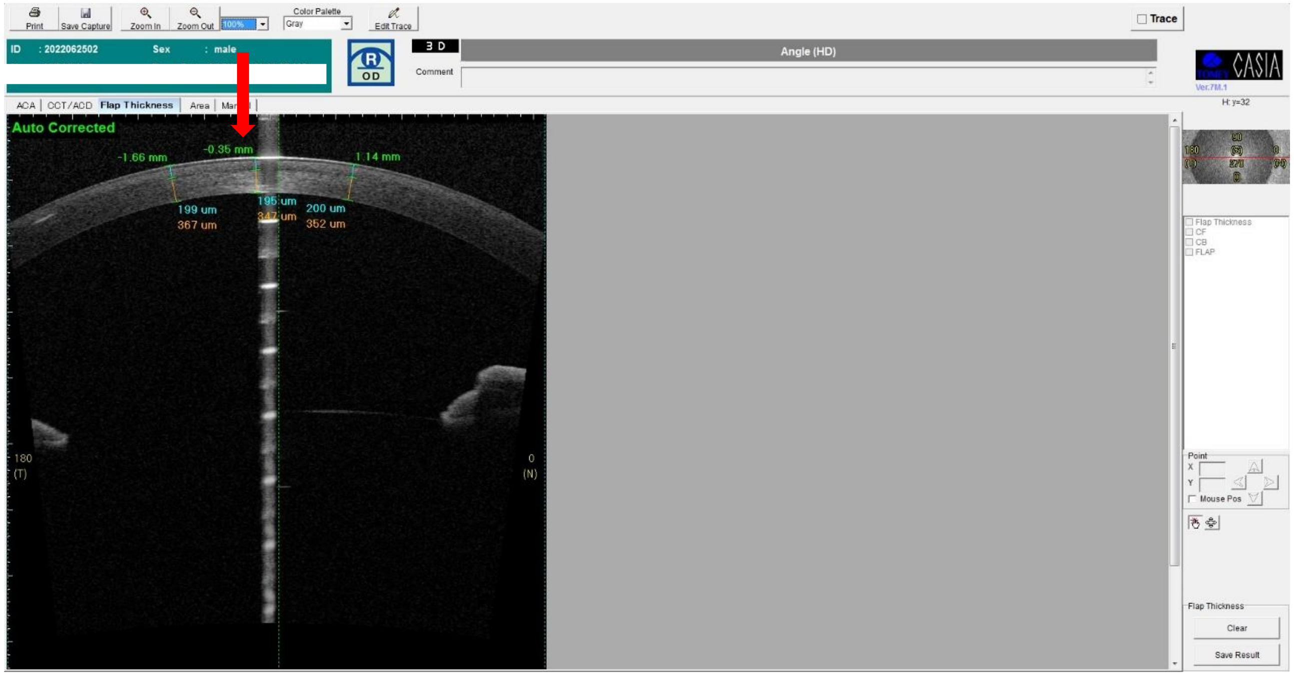

Print
Save Capture
Zoom In
Zoom Out
100%
Color Palette
Gray
Edit Trace
Trace
ID : 2022062502
Sex : male
OD
3 D
Angle (HD)
Comment
CASIA
ACA
OCT/ACD
Flap Thickness
Area
Auto Corrected
-1.66 mm
-0.35 mm
1.14 mm
195 um
199 um
367 um
200 um
352 um
180
(T)
0
(N)
Flap Thickness
CF
CB
FLAP
Point
Mouse Pos
Clear
Save Result

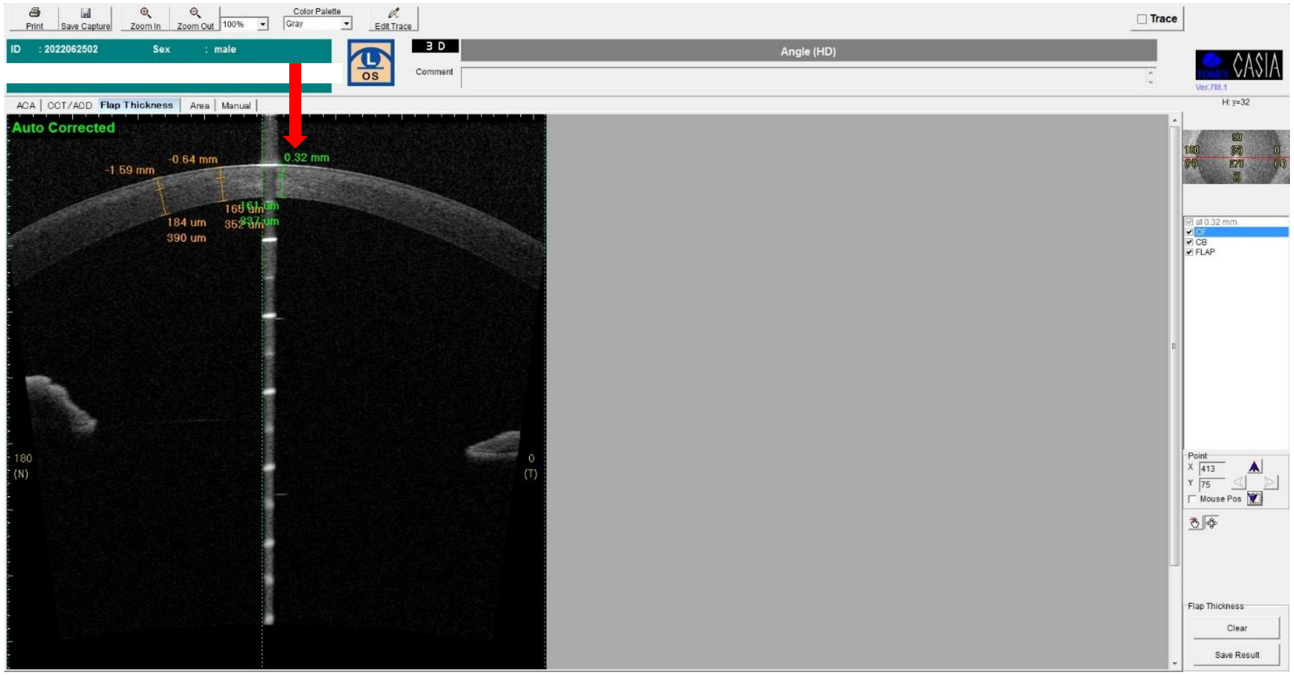

Print
Save Capture
Zoom In
Zoom Out
100%
Color Palette
Gray
Edit Trace
Trace
ID : 2022062502
Sex : male
OS
3 D
Angle (HD)
Comment
CASIA
ACA
OCT/ACD
Flap Thickness
Area
Manual
Auto Corrected
-1.59 mm
-0.64 mm
0.32 mm
184 um
390 um
at 0.32 mm
CF
CB
FLAP
180
(N)
0
(T)
Point
413
75
Mouse Pos
Flap Thickness
Clear
Save Result

N20

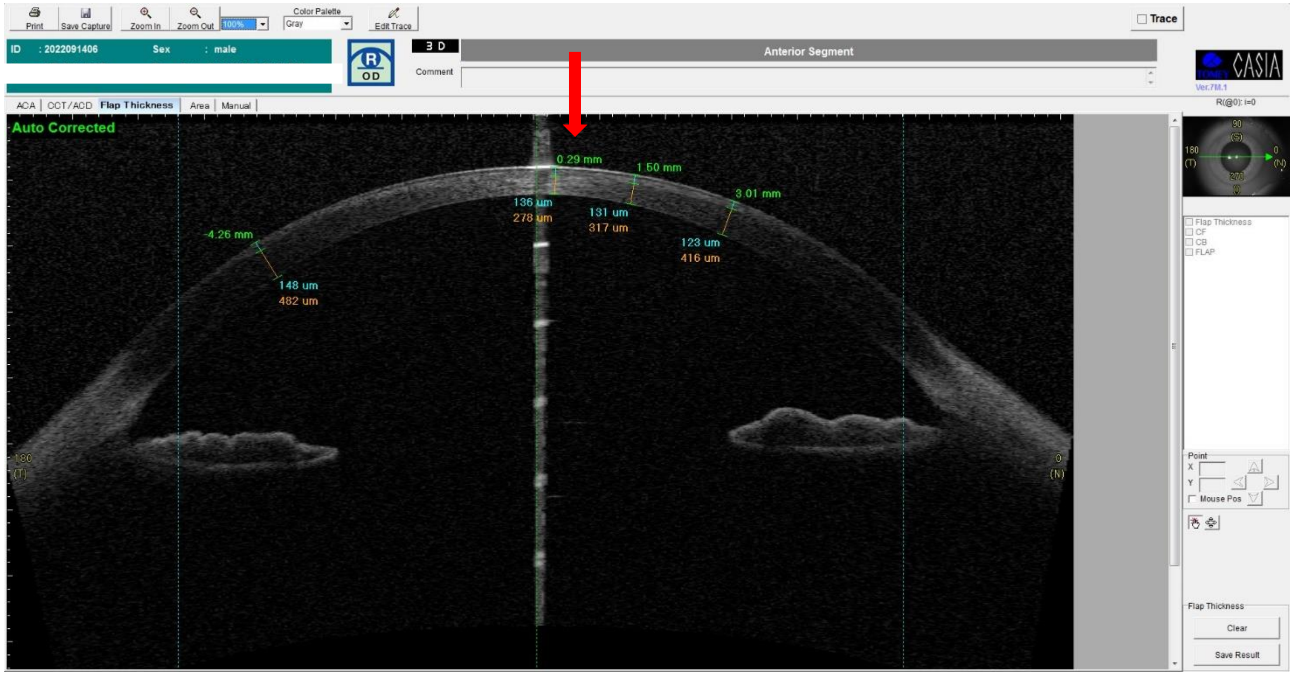

Print
Save Capture
Zoom In
Zoom Out
100%
Color Palette
Gray
Edit Trace
Trace
ID : 2022091406
Sex : male
R
OD
3 D
Anterior Segment
Comment
CASIA
Ver.7M.1
R(@0): i=0
ACA
OCT/ACD
Flap Thickness
Area
Manual
Auto Corrected
0.29 mm
1.50 mm
3.01 mm
136 um
278 um
131 um
317 um
123 um
416 um
4.26 mm
148 um
482 um
180
(T)
0
(N)
90
(S)
270
(I)
Flap Thickness
CF
CB
FLAP
Point
X
Y
Mouse Pos
Flap Thickness
Clear
Save Result

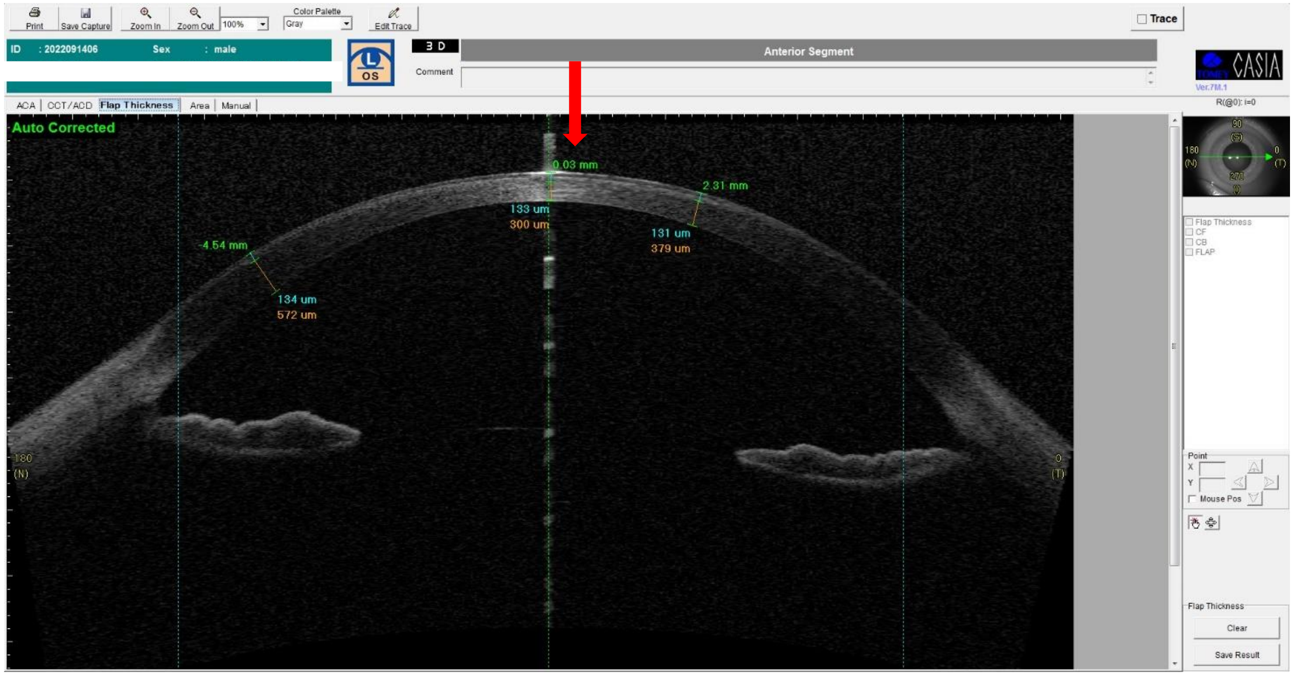

Print
Save Capture
Zoom In
Zoom Out
100%
Color Palette
Gray
Edit Trace
Trace
ID : 2022091406
Sex : male
L
OS
3 D
Anterior Segment
Comment
CASIA
Ver.7M.1
R(@0): i=0
ACA
OCT/ACD
Flap Thickness
Area
Manual
Auto Corrected
0.03 mm
2.31 mm
133 um
300 um
131 um
379 um
4.54 mm
134 um
572 um
180
(N)
0
(T)
90
(S)
270
(I)
Flap Thickness
CF
CB
FLAP
Point
X
Y
Mouse Pos
Flap Thickness
Clear
Save Result

N21

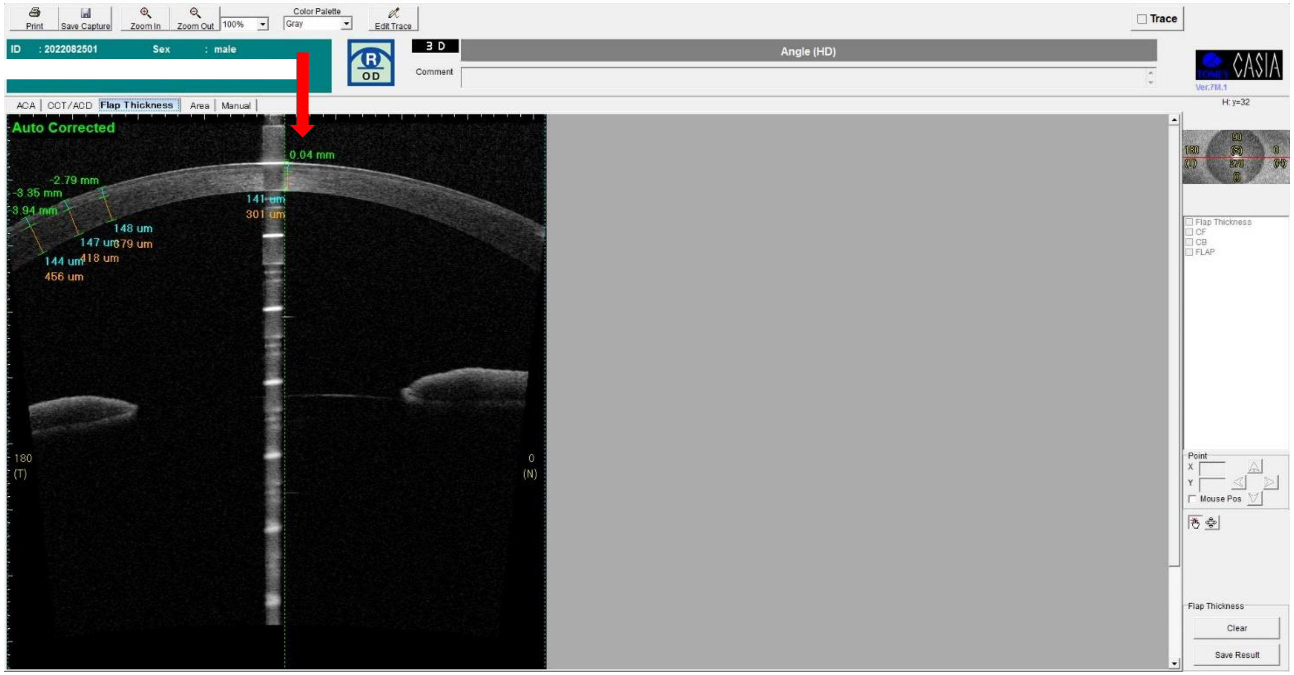

Print
Save Capture
Zoom In
Zoom Out
100%
Color Palette
Gray
Edit Trace
Trace
ID : 2022082501
Sex : male
R
OD
3 D
Angle (HD)
Comment
CASIA
Ver.7M.1
ACA
CCT/ACD
Flap Thickness
Area
Manual
Auto Corrected
0.04 mm
-2.79 mm
-3.35 mm
-3.94 mm
141 um
301 um
148 um
147 um
379 um
144 um
418 um
456 um
180
(T)
0
(N)
Flap Thickness
CF
CB
FLAP
Point
Mouse Pos
Clear
Save Result

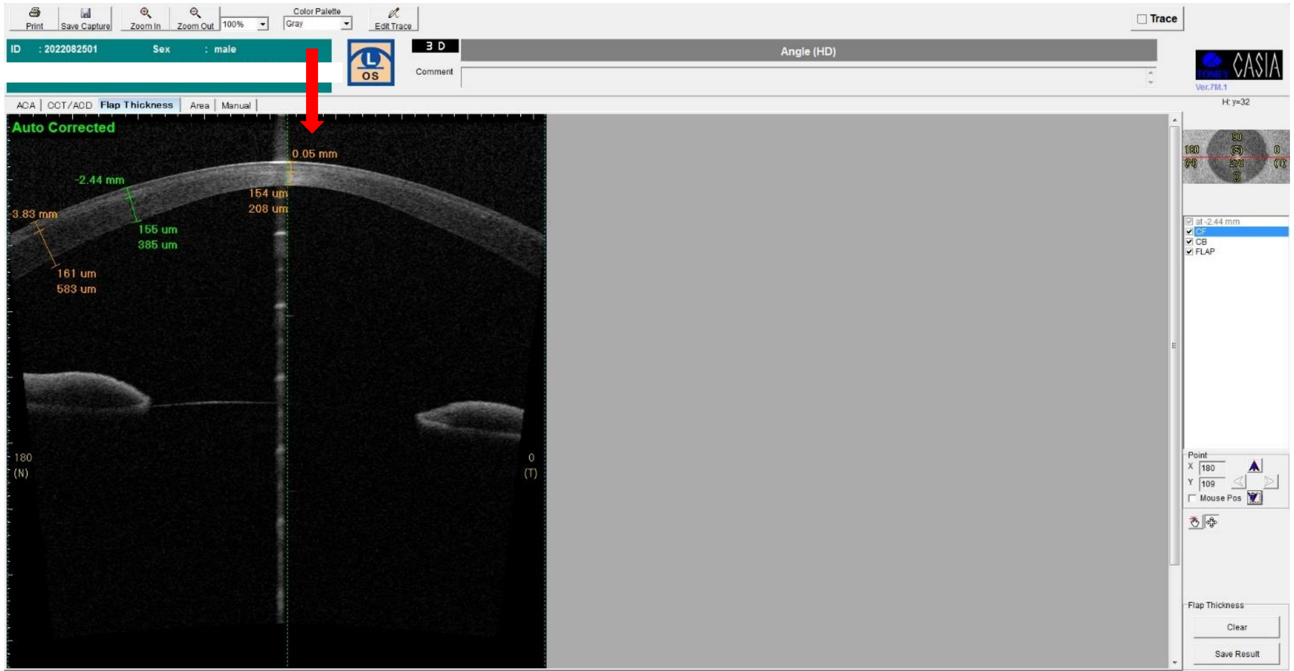

Print
Save Capture
Zoom In
Zoom Out
100%
Color Palette
Gray
Edit Trace
Trace
ID : 2022082501
Sex : male
L
OS
3 D
Angle (HD)
Comment
CASIA
Ver.7M.1
ACA
CCT/ACD
Flap Thickness
Area
Manual
Auto Corrected
0.05 mm
-2.44 mm
-3.83 mm
154 um
208 um
155 um
385 um
161 um
583 um
180
(N)
0
(T)
CF
CB
FLAP
Point
180
109
Mouse Pos
Flap Thickness
Clear
Save Result

N22

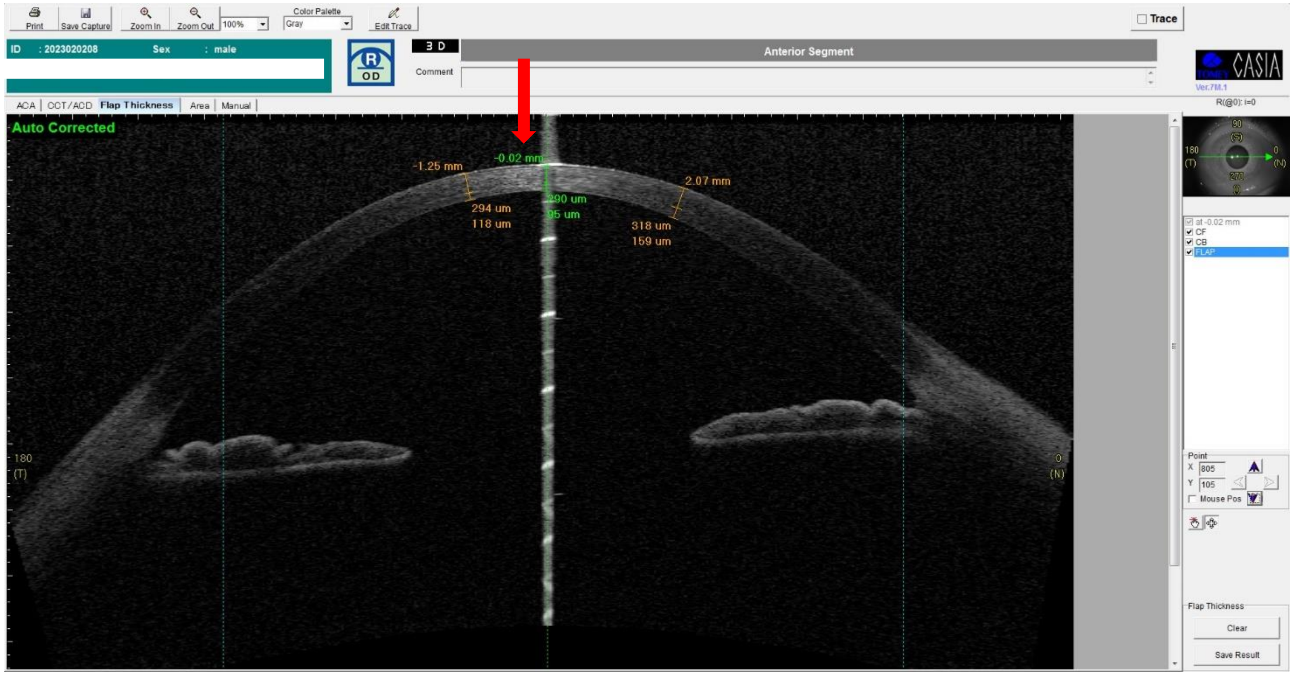

Print
Save Capture
Zoom In
Zoom Out
100%
Color Palette
Gray
Edit Trace
Trace
ID : 2023020208
Sex : male
R
OD
3 D
Anterior Segment
Comment
CASIA
Ver.7M.1
ACA
CCT/ACD
Flap Thickness
Area
Manual
Auto Corrected
-0.02 mm
-1.25 mm
2.07 mm
294 um
118 um
290 um
95 um
318 um
159 um
180
(T)
0
(N)
at -0.02 mm
CF
CB
FLAP
Point
805
105
Mouse Pos
Clear
Save Result

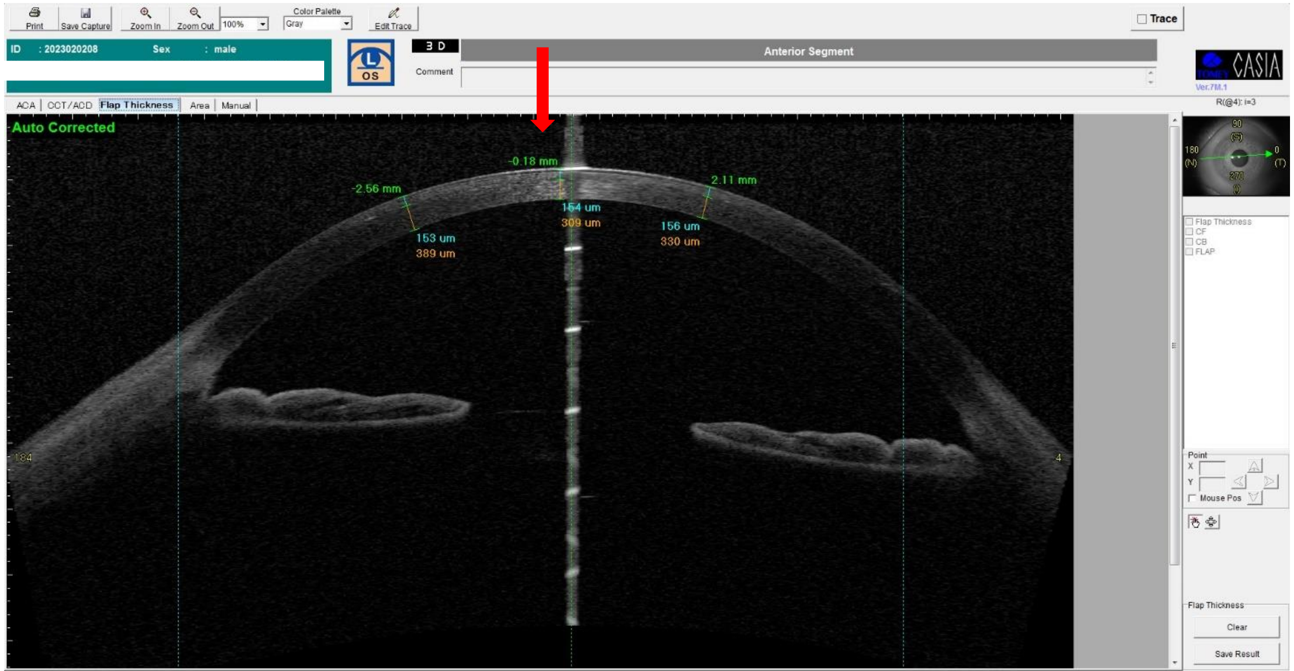

Print
Save Capture
Zoom In
Zoom Out
100%
Color Palette
Gray
Edit Trace
Trace
ID : 2023020208
Sex : male
L
OS
3 D
Anterior Segment
Comment
CASIA
Ver.7M.1
ACA
CCT/ACD
Flap Thickness
Area
Manual
Auto Corrected
-0.18 mm
-2.56 mm
2.11 mm
153 um
389 um
164 um
309 um
156 um
330 um
Flap Thickness
CF
CB
FLAP
Point
Mouse Pos
Clear
Save Result

N23

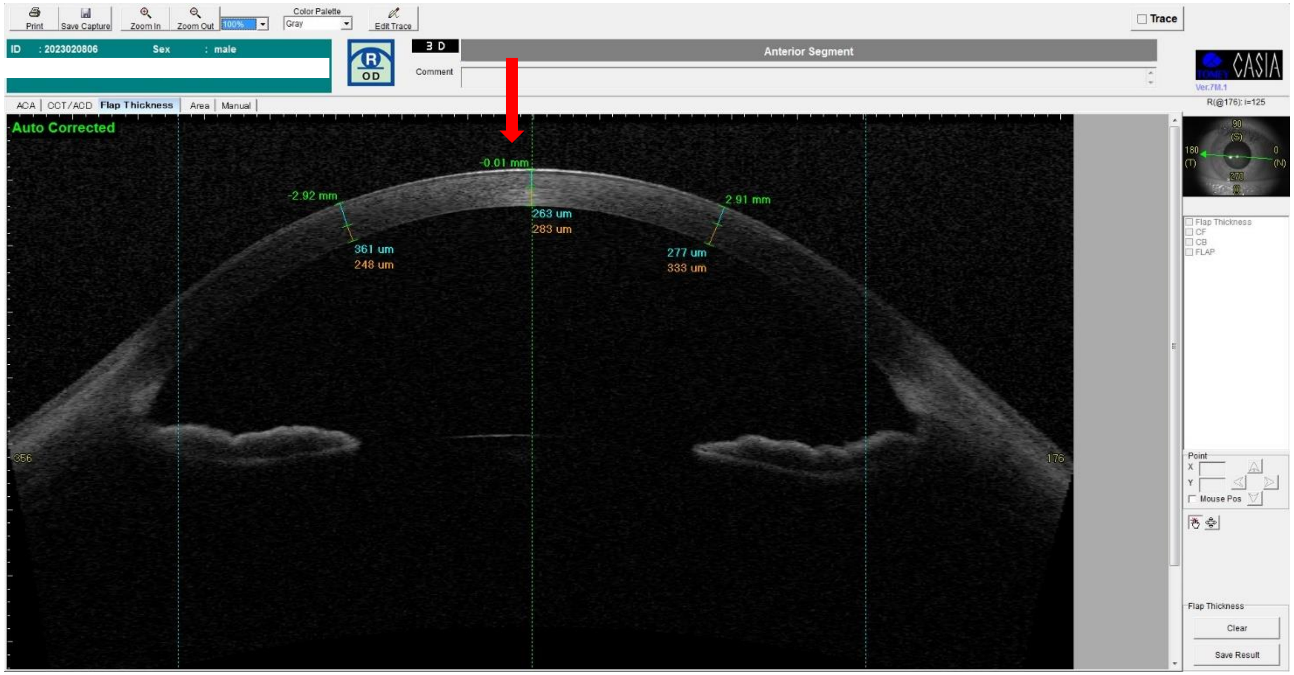
Anterior Segment
Auto Corrected
-0.01 mm
-2.92 mm
2.91 mm
263 um
283 um
361 um
248 um
277 um
333 um
Flap Thickness
Clear
Save Result

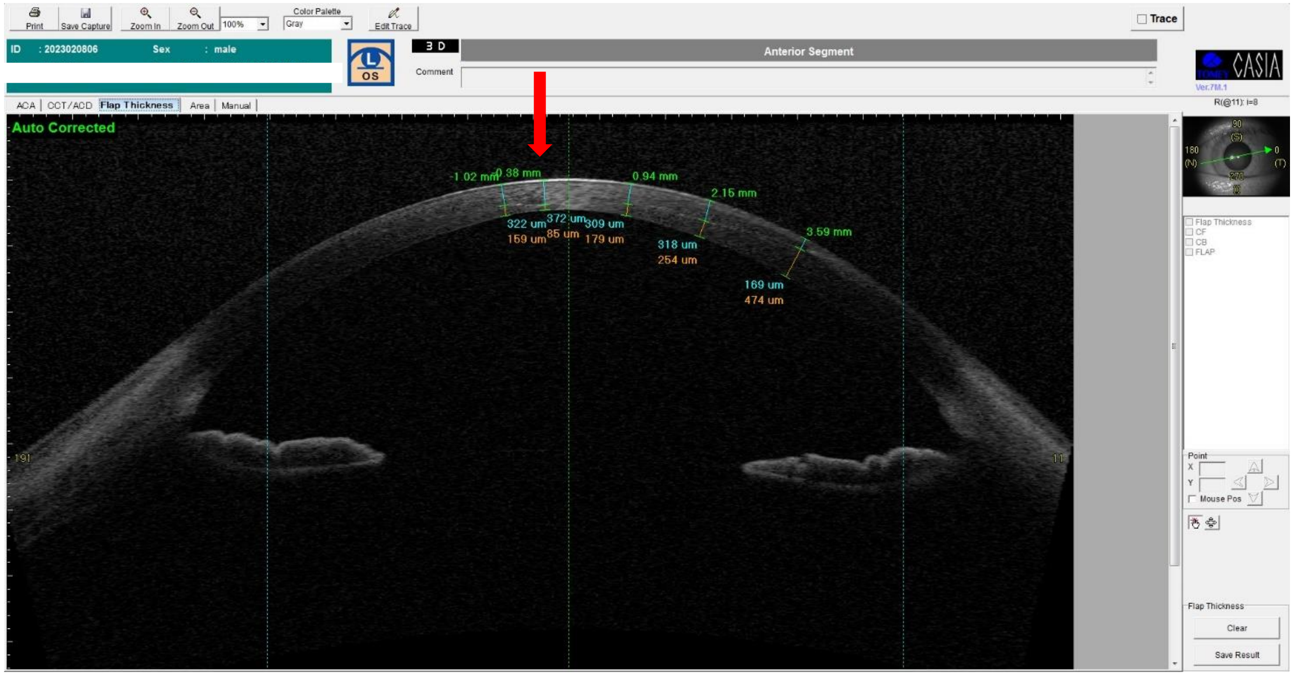
Anterior Segment
Auto Corrected
-1.02 mm
0.38 mm
0.94 mm
2.15 mm
3.59 mm
322 um
159 um
372 um
85 um
309 um
179 um
318 um
254 um
169 um
474 um
Flap Thickness
Clear
Save Result
